# Tween-20 Induces the Structural Remodeling of Single Lipid Vesicles

**DOI:** 10.1021/acs.jpclett.2c00704

**Published:** 2022-06-09

**Authors:** Lara Dresser, Sarah P. Graham, Lisa M. Miller, Charley Schaefer, Donato Conteduca, Steven Johnson, Mark C. Leake, Steven D. Quinn

**Affiliations:** † Department of Physics, University of York, York YO10 5DD, U.K.; ‡ Department of Electronic Engineering, University of York, York YO10 5DD, U.K.; § Department of Biology, University of York, York YO10 5DD, U.K.; ∥ York Biomedical Research Institute, University of York, York YO10 5DD, U.K.

## Abstract

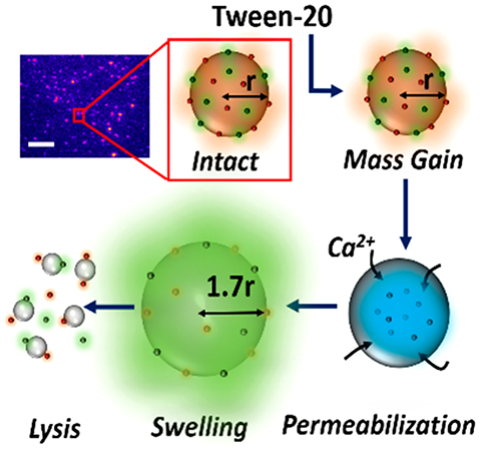

The solubilization of lipid membranes by Tween-20 is crucial for a number of biotechnological applications, but the mechanistic details remain elusive. Evidence from ensemble assays supports a solubilization model that encompasses surfactant association with the membrane and the release of mixed micelles to solution, but whether this process also involves intermediate transitions between regimes is unanswered. In search of mechanistic origins, increasing focus is placed on identifying Tween-20 interactions with controllable membrane mimetics. Here, we employed ultrasensitive biosensing approaches, including single-vesicle spectroscopy based on fluorescence and energy transfer from membrane-encapsulated molecules, to interrogate interactions between Tween-20 and submicrometer-sized vesicles below the optical diffraction limit. We discovered that Tween-20, even at concentrations below the critical micellar concentration, triggers stepwise and phase-dependent structural remodeling events, including permeabilization and swelling, in both freely diffusing and surface-tethered vesicles, highlighting the substantial impact the surfactant has on vesicle conformation and stability prior to lysis.

Tween-20 is a nonionic detergent widely used as a solubilizing agent of membrane proteins,^1,2^ for the inactivation of enveloped viruses,^3,4^ enhancing drug permeability,^5,6^ and regulating the diffusion of transmembrane proteins.^7^ However, despite such important roles across a number of applications, the mechanistic details of the Tween-20–membrane interaction remain largely ill-defined.

While the detergent is often used empirically as a general membrane solubilizer,^2^ experiments involving giant unilamellar vesicles (GUVs) have been employed to explore the underlying details. GUVs are highly controllable synthetic membrane mimetics that provide opportunities for interrogating detergent–membrane interactions in the absence of extraneous processes.^8^ High-intensity dark-field microscopy has previously enabled stepwise shrinkage, vigorous fluctuations, and bursting in 5 μm sized GUVs to be observed in response to Tween-20,^9^ and the observation of ∼10 μm sized vesicles revealed an increase in membrane surface area and transient and cyclic poration events strongly modulated by the surfactant concentration.^10,11^ When larger vesicles (>10 μm) were placed in a Tween-20 concentration gradient spanning 0–0.6 mM, the latter ∼10× the reported critical micellar concentration (CMC), the pore lifetime was found to be of the order of minutes.^12^ Optical microscopy also revealed that the opening of pores facilitated vesicle fusion, though whether oscillatory pore motion plays a role in this process remains unclear.^13^ Because of its ability to regulate membrane elasticity, Tween-20 has found utility in the production of highly pliable vesicles and niosomes that facilitate drug transport across the membrane.^14−16^ More recently, dynamic light scattering (DLS) and turbidity approaches have indicated that the bending rigidity of vesicle bilayers decreases quasi-exponentially with increasing concentration of the longer chain surfactant Tween-80.^17^ Thermodynamic approaches, including isothermal titration calorimetry, have also been employed extensively to evaluate the detergent CMC^18,19^ and to characterize the thermodynamics of the surfactant–membrane interaction.^11,20−22^ However, such approaches rely on ensemble averaging across the entire system and cannot report on individual vesicle conformation.

On the basis of these experimental results and others,^22−24^ a global three-step model has been proposed for the mode of Tween-20-induced solubilization. Here, the detergent monomers saturate the membrane in step 1, leading to the formation of mixed detergent–lipid micelles and fragmentation of the membrane in step 2 and the release of mixed detergent–lipid micelles to solution in step 3.^25^ A more quantitative extension of this model involves the formation of transient defects and micropores on the intact membrane prior to complete solubilization, where such events are dependent on the lipid composition, phase, and detergent concentration.^26^ However, the use of GUVs as model systems only represents one end of the membrane curvature space,^27^ and the use of conventional optical imaging approaches only allows for macroscopic changes in vesicle shape and packing density of GUVs typically >5 μm to be inferred. Consequently, such experiments provide little detail on the molecular level.^28^ Given highly curved nanoscopic vesicles, which have important implications in the context of biological trafficking,^29,30^ cannot be easily quantified by diffraction-limited optical microscopy techniques, it is important to explore the molecular details of the Tween-20 interaction at the opposite end of the membrane curvature space.

Recent developments in structural methods have brought the understanding of the molecular mechanisms of detergent-induced membrane disruption forward. For instance, single-vesicle Förster resonance energy transfer (FRET) imaging applied to submicrometer-sized vesicles revealed that the detergent Triton X-100 induces dynamic transitions between regimes.^27^ Similarly, vesicle swelling induced by the ionic detergent sodium dodecyl sulfate (SDS) was observed by the combined use of FRET, atomic force microscopy, and quartz crystal microbalance with dissipation (QCM-D) monitoring.^28^ Studies using a combination of light scattering, fluorescence correlation spectroscopy (FCS), cryo-electron microscopy, and coarse-grained molecular dynamic simulations have also revealed dynamic phase transitions and remodeling during the initial detergent–membrane interplay,^22,31−35^ suggesting the three-step model may also involve a number of additional, transient, and interlinked events.

Inspired by these insights, we interrogated the interaction between Tween-20 and large unilamellar vesicles (LUVs) of ∼200 nm diameter. While previous studies on GUVs utilized fluorescence imaging alone, we employed a range of tools, including QCM-D to explore mass and viscoelasticity changes, steady-state and time-resolved FRET spectroscopy to assess lipid partitioning, DLS and FCS to probe the hydrodynamic diameters of freely diffusing vesicles, and wide-field single-vesicle imaging tools to capture the response from immobilized vesicles. An important aspect of this work is the use of ensemble and single-vesicle FRET spectroscopy, which quantitatively reports on the distance between donor and acceptor probes embedded within the membrane with ∼1 nm spatial resolution. We previously used these techniques to quantify the solubilization of large unilamellar vesicles in response to Triton X-100 and SDS and reveal kinetically asynchronized reductions in FRET efficiency (reflecting vesicle swelling) and reduction in total fluorescence intensity (reflecting lysis).^27,28^ Here, we used these techniques to explore the structural integrity of vesicles composed of POPC (1-palmitoyl-2-oleoylglycero-3-phosphocholine) in response to Tween-20, and we extract information about the membrane composition and interaction by applying a mass-action model to variations in the FRET efficiency.^36^ To further characterize the interaction, we also implemented an ultrasensitive approach to quantify the extent of membrane disruption by Tween-20 whereby vesicles filled with the fluorescent calcium indicator Cal-520 report on Ca^2+^ entry into vesicles as a consequence of permeabilization.^37^ Our discovery of the structural remodeling of both freely diffusing and surface-immobilized vesicles in response to Tween-20, even at concentrations below the CMC, contributes new clues to the underlying solubilization mechanism, and we expect the presented tools to have far-reaching applications in elucidating the underlying membrane damage mechanisms associated with a wide variety of membrane disruptive molecules.

QCM-D was first used to examine time- and concentration-dependent changes in the mass and viscoelasticity of surface-immobilized LUVs in response to Tween-20. The LUVs were modified with 1 mol % of biotinylated lipids and were tethered via NeutrAvidin to a bovine serum albumin (BSA)-coated SiO_2_ sensor containing 2 mol % biotinylated-BSA (BSA-Bi), as demonstrated by real-time changes in the resonance frequency shift (Δ*F*) and energy dissipation (Δ*D*), reflecting the mass and viscoelasticity of the surface, respectively (Figure 1A). After the immobilization procedure, the sensor surface was rinsed with buffer to remove unbound vesicles. Upon addition of 0.02 mM Tween-20, we then observed a 10 Hz reduction in Δ*F* reflecting an increase in the sensor mass, concurrent with a 20% increase Δ*D*, representing an increase in viscoelasticity (Figure 1A). Control experiments performed simultaneously by using sensors coated in BSA, biotinylated-BSA, and NeutrAvidin but lacking vesicles displayed similar Δ*F* and Δ*D* responses when flushed with Tween-20, which we attributed to a combination of the buffer change and mass increase caused by surfactants nonspecifically binding to the surface (Figure S1). However, when the control surface was washed with buffer, a recovery in Δ*F* and Δ*D* to similar levels prior to the introduction of Tween-20 was observed, indicating the detergent does not lead to the release of BSA-Biotin or NeutrAvidin, and the nonspecific attachment of Tween-20 is reversible. The rate of change across both signals was approximately three times faster when vesicles were present, pointing toward a more rapid mass and viscoelastic gain. The subsequent and dramatic increase in Δ*F* at ∼110 min, as seen on the vesicle-coated surface (Figure 1A), and the anticorrelated decrease in Δ*D* are then explained by the removal of material and surfactant from the substrate with a rate constant of 0.25 ± 0.01 Hz min^–1^. Similar behavior was observed when 0.04 and 0.06 mM Tween-20 were flushed across vesicle-coated surfaces (Figure 1B,C), indicating similar degrees of mass gain and comparable solubilization rates (0.27 ± 0.01 Hz min^–1^). Under the latter conditions we observed a further reduction in Δ*F* and a corresponding increase in Δ*D* after the major mass loss event that we assigned to the adsorption of mixed detergent–lipid micelles on the surface. The Tween-20–vesicle interaction was also visualized by plotting changes in Δ*F* against Δ*D* across the experiment, with the interaction defined as complete when Δ*F*/Δ*t* < 2 Hz/10 min (Figure 1D). Under each condition tested, the Δ*F* versus Δ*D* responses displayed an initial turning point, reflecting a mass gain and an increase in viscoelasticity, followed by a substantial mass loss to the solution, which we assigned to lysis. Taken together, the QCM-D data indicate that the initial deposition of Tween-20 onto the vesicle surface triggers a structural remodeling event that precedes the loss of material to the solution. A surprising outcome of this analysis is that the initial structural rearrangement occurred at concentrations of detergent below the CMC (∼0.06 mM), pointing toward an interaction between detergent monomers and vesicles that triggers substantial conformational changes prior to lipid release.

**Figure 1:**
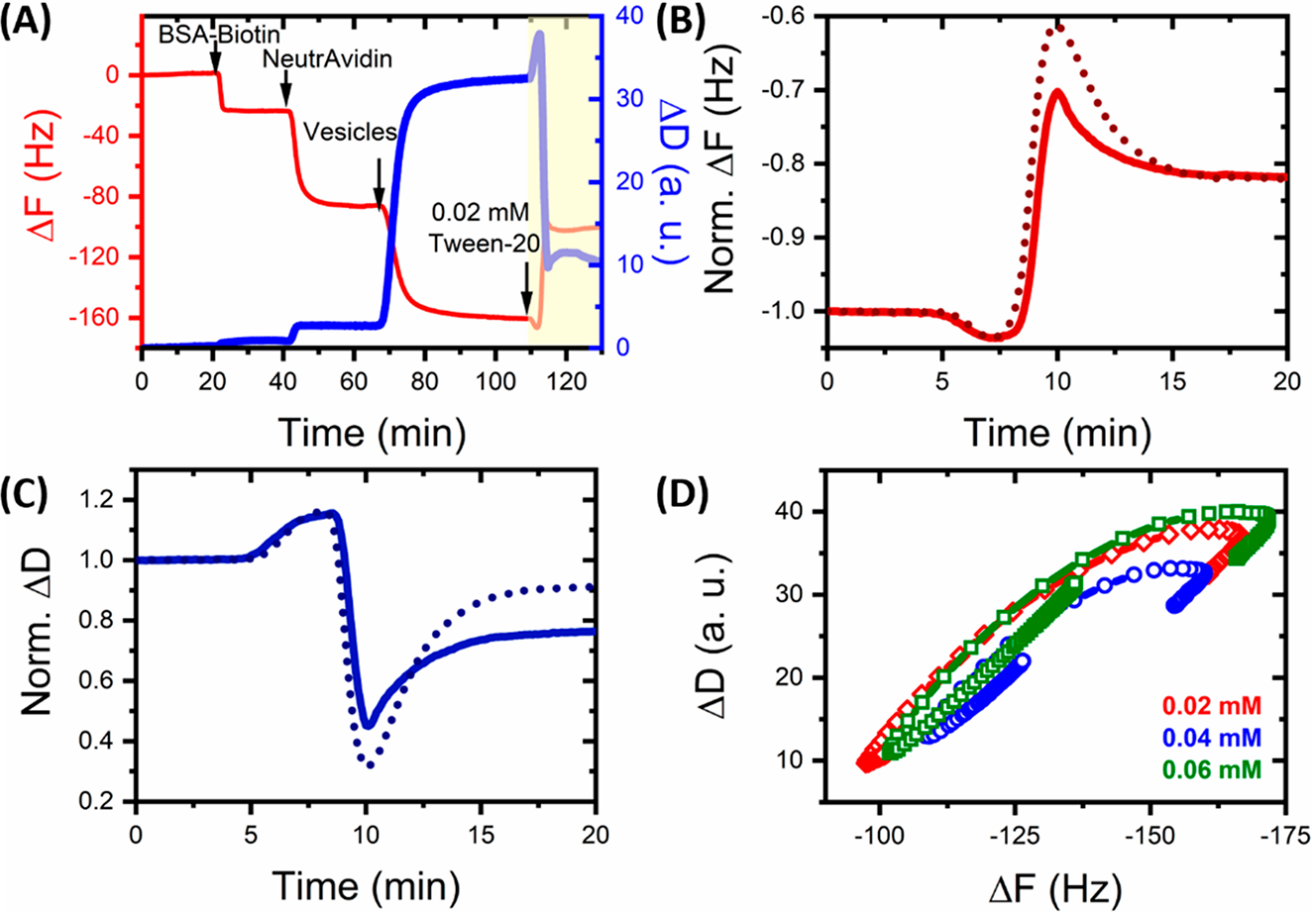
QCM-D of Tween-20 interactions with surface-tethered vesicles. (A) Evolution of Δ*F* (red) and Δ*D* (blue) (seventh harmonic) upon the addition of LUVs to a BSA-biotin- and NeutrAvidin-coated surface. Addition of Tween-20 at 0.02 mM (yellow shaded area) was performed after washing the vesicle-saturated surface with 50 mM Tris buffer (pH 8). Normalized variations in (B) Δ*F* and (C) Δ*D* observed in response to 0.04 mM (solid lines) and 0.06 mM (dashed lines) Tween-20 injected at *t* = 5 min. (D) Frequency versus dissipation observed during the interaction between surface immobilized vesicles and Tween-20.

To gain molecular-level insights into the structural changes, we next performed steady-state and time-resolved FRET measurements, recording variations in fluorescence intensity and lifetime from freely diffusing vesicles incorporating the lipophilic probes DiI and DiD (Figure 2A). The incorporation of 0.1 mol % DiI (donor) and 0.1 mol % DiD (acceptor) into LUVs produces a FRET efficiency per vesicle, *E*, of ∼0.5, enabling structural variations such as swelling or compaction to be reported via changes to *E* in either direction. As shown in Figure 2B, changes in the fluorescence emission spectra after the addition of Tween-20 are substantial: we observed a 2-fold increase in the peak DiI fluorescence intensity concurrent with a loss of sensitized DiD emission as Tween-20 was progressively titrated, translating to a reduction in *E* and an increase of ∼58% in the mean donor–acceptor separation distance (Figure 2B). Vesicle solubilization by Tween-20 was more prevalent at higher temperatures, as revealed by the shift of the half-maximal concentration to lower values. Previously, we quantified this by curve-fitting the Hill model^27^ to the data, which yielded a half-maximal concentration of 0.11 ± 0.01 mM at 4 °C, ∼0.04–0.06 mM at 21 °C (similar to the reported CMC^19^), and 0.03 ± 0.01 mM at 37 °C. The Hill coefficient was 4.1 ± 0.9 and independent of temperature. From these features we extracted thermodynamic information about the surfactant–lipid interactions by adopting a mass-action model (Supplementary Text 1, Figure S2), which predicts that the sharpness of the FRET efficiency curve is controlled by the excess of free surfactants near the solubilization concentration. The model further describes the temperature-controlled shift of the solubilization concentration in terms of a surfactant-to-lipid-membrane binding energy of 31 ± 3 kJ/mol, which is modified for the increasing surfactant-to-lipid ratio in the membrane at increasing concentrations by using a Flory–Huggins free energy of mixing. From our curve fits we extracted a near-athermal Flory–Huggins parameter, χ, of 1.2 ± 0.2 (Table S1). As χ = 0 indicates ideal miscibility and χ > 2 complete incompatibility, χ ≈ 1.2 suggests favorable interactions between Tween-20 monomers within the membrane, and we speculate that the surfactant might dynamically cluster to locally disrupt the bilayer and lead to pore formation. Taken together, the overall change in FRET efficiency is consistent with an increase in the average spatial separation of the probes and was assigned to vesicle expansion and/or micellization.

**Figure 2:**
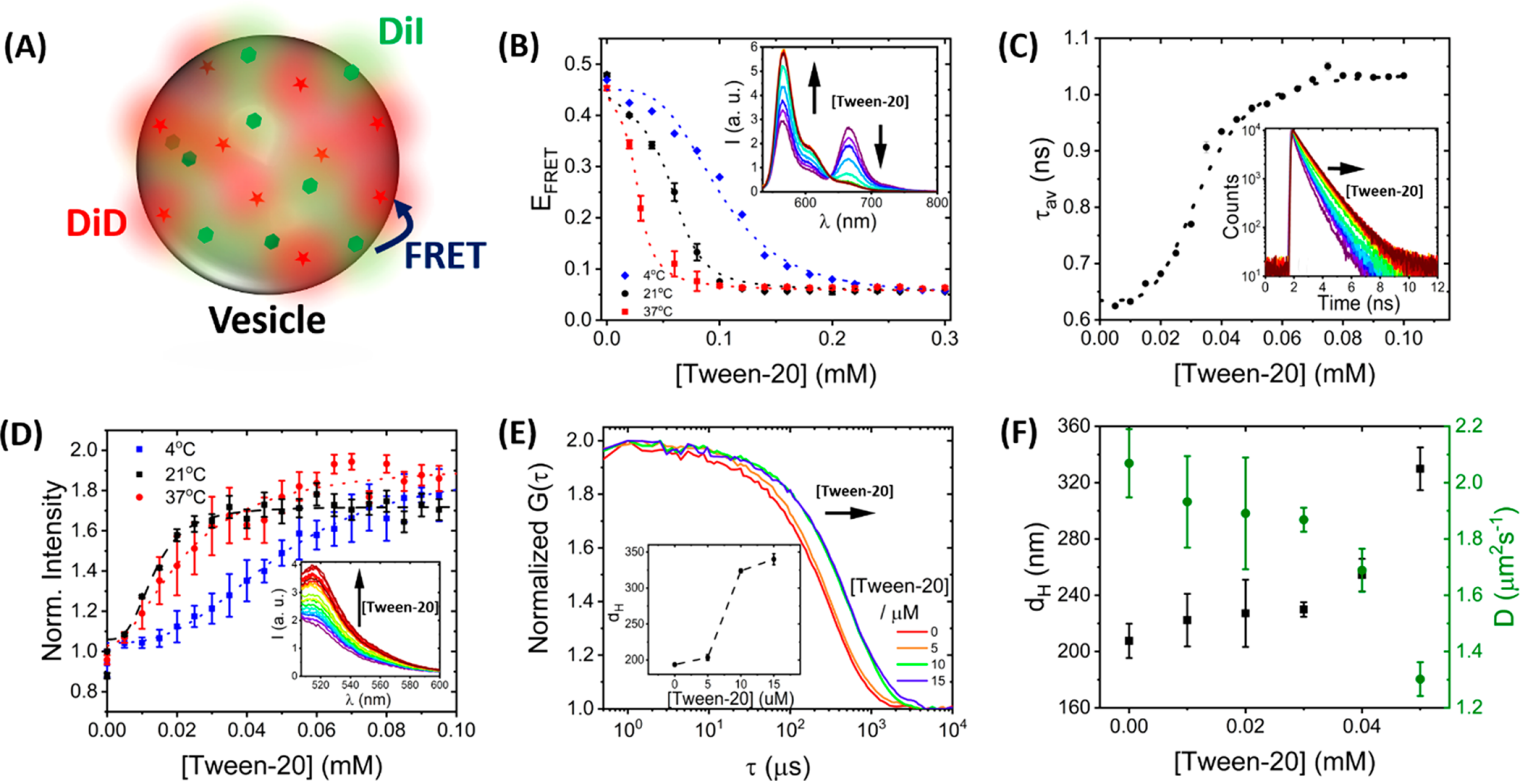
Tween-20 Induces the structural remodelling of freely diffusing vesicles. (A) Schematic of LUVs containing 0.1% DiI and 0.1% DiD. (B) Ensemble FRET efficiency of LUVs at 4 °C (blue), 21 °C (black), and 37 °C (red) as a function of Tween-20. Dashed lines correspond to mass-action fits. Inset: corresponding variation in fluorescence emission spectra. (C) Average lifetime of DiI as a function of Tween-20 at 21 °C. Inset: corresponding time-resolved fluorescence decays and instrumental response function (gray). (D) Tween-20 stimulated calcium responses of LUVs encapsulating Cal-520 at 4 °C (blue), 21 °C (black), and 37 °C (red). Inset: corresponding variation in fluorescence emission spectra. (E) Normalized variation in the DLS correlation curves obtained from LUVs versus Tween-20. Inset: variation in the mean hydrodynamic diameters, *d*_H_. (F) Variation in *d*_H_ (black) and diffusion coefficient (green) of single vesicles reported by FCS.

To confirm an energy transfer mechanism, we evaluated the fluorescence lifetime of DiI in the presence of DiD. The amplitude-weighted average lifetime, τ, progressively increased with Tween-20 concentration, consistent with a progressive enhancement of the donor intensity and corresponding decrease in *E* (Figure 2C). The decays were best fitted to a triexponential model after reconvolution with the instrument response function (Figure S3) likely representing vesicles with coexisting phases and membrane asymmetry.^38−41^ In the absence of Tween-20 (21 °C), we recorded τ = 0.62 ± 0.01 ns, representative of quenched DiI. At 0.1 mM Tween-20, τ increased to 1.03 ± 0.01 ns, with the lifetime components increasing by >30% of their initial values (Figure 2C). At 4 °C, the initial lifetime was 4-fold longer, likely representing an increase in the fraction of phase-sensitive components, and a 2-fold increase in τ was observed across the titration (Figure S4). At 37 °C, we observed similar behavior with a half-maximal concentration of 0.02 mM, comparable to Figure 2B (Figure S4). These data also point toward fluorophore separation, though whether this was due to expansion and/or micellization required further investigation.

We next applied an approach based on the measurement of Ca^2+^ entry into vesicles to quantify the magnitude of membrane permeabilization and assess solution exchange between the vesicle interior and exterior. Here, we used LUVs encapsulating Cal-520, whose fluorescence emission intensity increases upon binding Ca^2+^. In the context of protein-induced membrane permeabilization,^37,42^ Ca^2+^ flux into vesicles triggers a local Ca^2+^ concentration increase per vesicle, yielding a concentration-dependent increase in Cal-520 emission. Nonencapsulated molecules were removed by size exclusion chromatography (Figure S5), and as Tween-20 was titrated (21 °C), a 2-fold increase in the peak Cal-520 intensity was observed (Figure 2D). The Cal-520 signals minus Tween-20 were invariant and monoexponentially increased (*k* = 0.10 ± 0.01 s^–1^) after surfactant addition (Figure S6). To estimate Ca^2+^ influx into LUVs, we also added the cation transporter ionomycin, enabling the relative magnitude of Tween-20 induced Ca^2+^ influx to be inferred. The intensity enhancement at 0.06 mM Tween-20 was comparable to that observed with 1 mg/mL ionomycin (Figure S7), yielding a relative Ca^2+^ influx of ∼95%. Control experiments indicated that (i) Ca^2+^ did not cross the membrane in the absence of Tween-20 (Figure S7), (ii) Ca^2+^ did not induce vesicle fusion, as has been observed previously for negatively charged vesicles^43,44^ (Figure S8), and (iii) the Cal-520 fluorescence is insensitive to Tween-20 (Figure S9), providing confidence that the observed enhancements are due to surfactant-induced membrane permeabilization. At 4 and 37 °C the measured Ca^2+^ influxes after Tween-20 addition were 98% and 96%, respectively (Figure 2D). We note that Hill models applied to the Cal-520 enhancements revealed half-maximal constants generally lower than those observed in Figure 2B (Table S2), implying membrane permeabilization precedes lipid separation in the ensemble.

To establish whether the fluorescence signals were correlated to changes in vesicle morphology, we performed DLS to assess the hydrodynamic diameters (*d*_H_) of LUVs as previously described.^27,28,45,46^ Freshly prepared vesicles exhibited *d*_H_ = 194 ± 2 nm with a polydispersity index of ∼0.47 (Figure 2E and Figure S10), and in low concentrations (<15 μM) of Tween-20, the correlation curves progressively shifted toward longer lag times. Assuming spherical vesicles, this translates to a 75% increase in vesicle size, attributed to vesicle expansion, fusion, or the combination of both given the polydispersity index increased toward 20 μM (Figure S10).

To minimize fusion and test for expansion, we investigated the hydrodynamic diameters using FCS. Unlike DLS, FCS is used on systems in which the concentration of fluorescent species is subnanomolar; hence, they negligibly interact. The diffusion coefficients, *D*, and hydrodynamic diameters of LUVs containing 0.1% DiI were recovered from the correlation functions obtained from vesicles freely diffusing through a confocal volume (Figure 2F and Figure S11). Freshly prepared vesicles displayed *d*_H_ = 208 ± 12 nm and *D* = 2.1 ± 0.1 μm^2^ s^–1^. *d*_H_ then increased to ∼330 nm in 0.06 mM Tween-20, corresponding to a reduction in *D* to 61% of its initial value, confirming expansion within single intact vesicles. The relative increase in *d*_H_ is comparable to LUVs of similar composition in low concentrations of Triton X-100, which we interpreted as evidence that Tween-20 leads to a similar degree of vesicle perturbation at the detergent CMC.

To further explore the structural changes, we performed single-vesicle FRET imaging by using a wide-field, objective-based total internal reflection fluorescence microscope that enables the parallel imaging of DiI and DiD emission.^28,47^ Here, the mean FRET efficiencies of single surface-immobilized vesicles labeled with 0.1% DiI and 0.1% DiD were monitored upon addition of Tween-20. Surface immobilization was achieved by incorporating 1% biotinylated lipids into the LUVs for coupling to a glass coverslip via biotin–NeutrAvidin interactions (Figure 3A). An oxygen scavenger cocktail consisting of glucose oxidase, catalase, and Trolox was also added to the imaging buffer to minimize photobleaching and photoblinking.^48,49^ In the absence of Tween-20, ∼150–200 vesicles per 25 × 50 μm^2^ field were imaged, representing surface-tethered vesicles separated by a nearest-neighbor distance of ∼1 μm (Figure 3B). When imaged under low excitation powers (<8.2 mW cm^–2^), the donor, acceptor, and FRET trajectories remained photostable over a 50 s time window (Figure 3C). Because of efficient FRET between DiI and DiD, fluorescence was observed across both donor and acceptor detection channels, and changes to these intensities after addition of Tween-20 were recorded. As Tween-20 was added, we observed a substantial decrease in the acceptor emission and a corresponding increase in the donor emission because of reduced FRET between the probes (Figure 3C). Upon addition of surfactant at 0.06 and 0.12 mM, the number of fluorescent spots per field of view, the mean total emission intensity per vesicle defined as ⟨*I*_T_⟩ = ⟨*I*_D_ + *I*_A_⟩ (where *I*_D_ and *I*_A_ are the donor and acceptor intensities), and the nearest-neighbor vesicle separation distance, ⟨*d*⟩, remained largely unchanged (Figure 3D), indicating the presence of intact vesicle structures. At higher detergent concentrations, a 2-fold increase in ⟨*d*⟩ and a corresponding decrease in ⟨*I*_T_⟩ were observed, indicating removal of material from the surface. The FRET efficiency drop observed across individual vesicles at low Tween-20 concentrations (Figure 3E) could not therefore be attributed to lipid loss or partial vesicle detachment. Instead, this observation implies a structural change, namely expansion, taking place within individual vesicles, with minimal loss of lipid material to solution. This result is opposed to previous observations where Tween-20 had no effect on the modal size distribution of human-derived extracellular vesicles (EVs);^50^ however, an explanation for this discrepancy rests in the lipid composition. In this work we used model membranes composed of POPC lipids, whereas EVs are enriched in cholesterol.^51^ As previously demonstrated, PC vesicles composed of modest cholesterol content resist detergent-induced solubilization, likely due to cholesterol obstructing the initial step of detergent molecules inserting into the lipid bilayer.^27^ In contrast to EVs, >95% of vesicles investigated here exhibited the swelling behavior. When the Tween-20 concentration was then increased toward 0.3 mM, ⟨*d*⟩ also progressively increased to 2.6 ± 0.2 μm, corresponding to a reduction in the number of vesicles per field of view which we assigned to the removal of a fraction of the vesicle population (Figure 3D). Those that remained on the surface displayed a stepwise shift toward lower FRET efficiency signatures indicative of further expansion.

**Figure 3:**
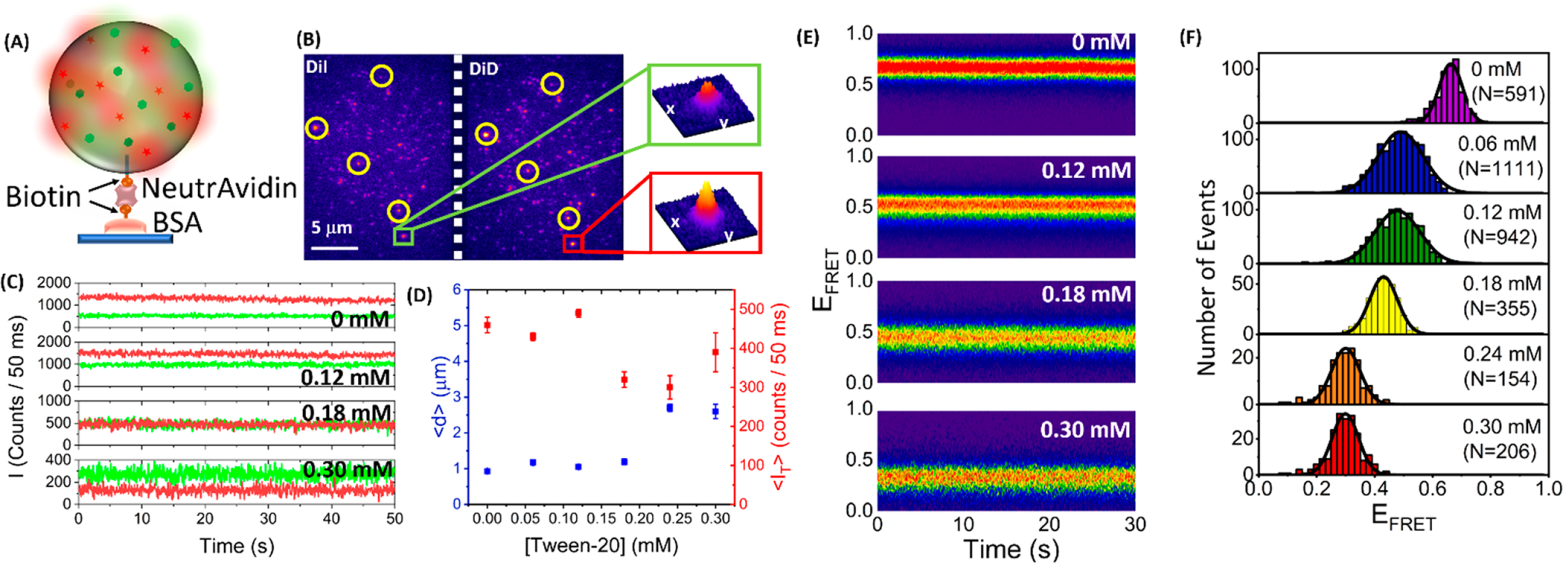
Single-vesicle imaging of surface-tethered LUVs. (A) Schematic of the immobilization scheme. LUVs incorporating 1% Biotin-PE were attached to a glass coverslip via BSA by using biotin–NeutrAvidin chemistry. (B) Representative TIRF image obtained from surface-tethered LUVs in the absence of Tween-20, showing representative acceptor signals colocalized with their corresponding donors (yellow circles). Insets: 3D intensity plots of DiI and DiD emission from a single surface-immobilized vesicle. (C) Representative DiI (green) and DiD (red) intensity traces from individual LUVs in the absence and presence of surfactant. (D) Variation in ⟨*d*⟩ and ⟨*I*_T_⟩ obtained as a function of Tween-20. (E) Contour plots of the time evolution of the FRET population as a function of Tween-20. Contours are plotted from blue (lowest population) to red (highest population). (F) Histograms of the mean FRET efficiency were obtained from single immobilized vesicles after incubation with Tween-20-rich solutions. Solid black lines represent Gaussian fits.

Histograms of the mean energy transfer efficiencies were generated from several hundred single vesicles per condition, allowing for discrete conformational changes to be verified (Figure 3F). The distributions displayed Gaussian behavior, and in the absence of Tween-20, a peak efficiency of 0.66 ± 0.08 was recorded, corresponding to intact vesicles where the dyes are spatially separated close to their Förster radius. With increasing Tween-20 concentrations toward 0.18 mM, the FRET population decreased in a stepwise manner, indicative of a 35% increase in the mean dye-pair separation distance. At concentrations >0.24 mM, the peak position shifted further to 0.30 ± 0.09. Overall, this observed reduction agrees well with the conformational changes reported by QCM-D, DLS, and FCS and further confirms that Tween-20 induces the structural remodeling of single surface-tethered vesicles.

To probe the size distribution of LUVs in response to Tween-20, we also employed low-voltage scanning electron microscopy (SEM),^52^ where micrographs revealed that freshly prepared LUVs were predominantly spherical (circularity = 0.68 ± 0.01) with a mean diameter of 72 ± 3 nm (Figure 4A). In the presence of Tween-20 at concentrations similar to those used in Figure 3, the circularity was similar (0.67 ± 0.01) (Figure 4B,C); however, the size distribution substantially broadened (Figure 4D). While our SEM sample preparation utilized a thin (5 nm) conductive layer, which as previous studies indicate does not substantially alter the morphology,^53^ we note that the requirement to dehydrate the vesicles may explain why the observed size in the absence of detergent is lower than those reported by FCS and DLS. Nevertheless, our observations of spherical morphologies are in line with similar studies^54,55^ and taken in conjunction with our fluorescence and QCM-D data, the results are broadly supportive of Tween-20 induced vesicle swelling.

**Figure 4:**
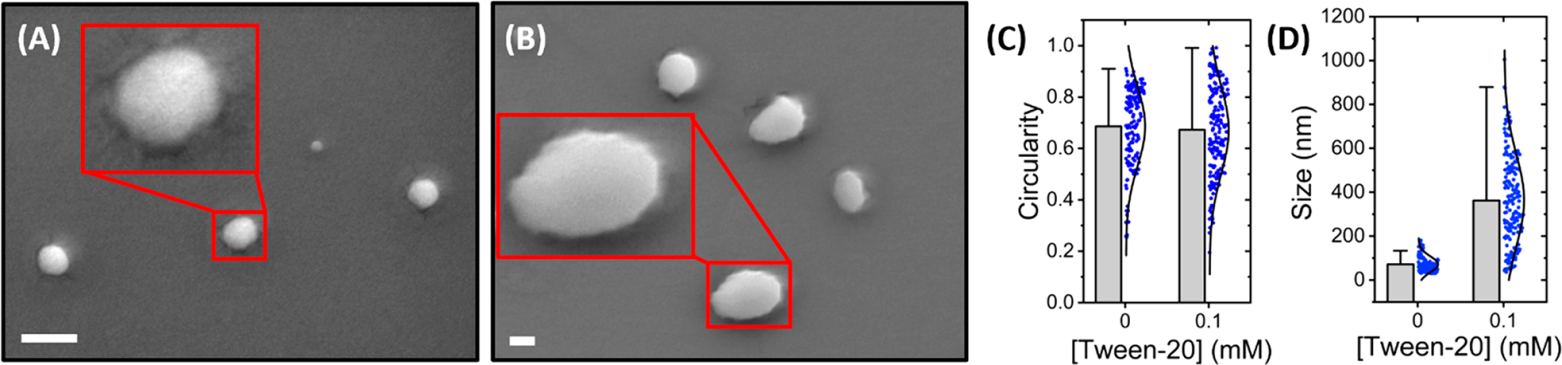
SEM analysis of single LUVs. Micrographs of vesicles (A) in the absence and (B) presence of 0.1 mM Tween-20. Scale bars = 100 nm. Also shown are bar plots summarizing the variation in (C) circularity and (D) particle size for vesicles in the absence (*N* = 137) and presence (*N* = 176) of Tween-20.

All measurements discussed point toward the global remodeling of vesicles in response to Tween-20. However, whether the interaction also involved content leakage via membrane permeabilization remained an open question. To assess this, we investigated membrane integrity during interaction with Tween-20 by monitoring the influx of calcium into single LUVs encapsulating Cal-520. We incubated immobilized vesicles in buffer containing 10 mM Ca^2+^ and imaged them under TIRF conditions with and without Tween-20. We imaged 10 fields of view per condition, allowing us to quantify the intensity distributions from several thousand vesicles, before adding buffer solutions rich in Tween-20 and Ca^2+^. If the Ca^2+^ entry occurred, an increase in intensity per vesicle was detected (Figure 5A), leading to shifts in the distribution toward higher intensity values. In the absence of Tween-20 and Ca^2+^, ∼100–150 vesicles per field were identified (Figure 5B), and the intensity distribution displayed log-normal behavior with a peak of 85 counts/100 ms, which is to be expected for diffusing molecules^56^ (Figure 5E). When 10 mM Ca^2+^ was introduced, the number of spots remained unchanged (Figure 5C), and the intensity distribution was comparable (91 counts/100 ms) (Figure 5E), indicating negligible levels of Ca^2+^ influx. However, with 0.01–0.04 mM Tween-20, we observed a clearly discernible difference, with the vesicle spots appearing progressively brighter (Figure 5D), leading to a transition from low-to-high shifts in the intensity population distributions (Figure 5E and Figure S12). At concentrations >0.04 mM, we then observed a shift in the peak position toward lower values, which was assigned to Cal-520 leakage. In all cases, a 2-fold or greater increase in Cal-520 intensity was observed, in line with our ensemble measurements and those observed by others.^37^ Importantly, the fluorescence signals before and after addition of Tween-20 were stable (Figure S12), and the number of surface-immobilized vesicles remained invariant as Tween-20 was flushed across the surface. The combined data thus support an interaction between Tween-20 and LUVs that involves swelling, membrane permeabilization, and solution exchange between intact vesicles and the local environment. Despite only moderate changes in the Cal-520 intensity, this analysis suggests substantial permeabilization, given a doubling of intensity was also observed in the presence of ionomycin.

**Figure 5:**
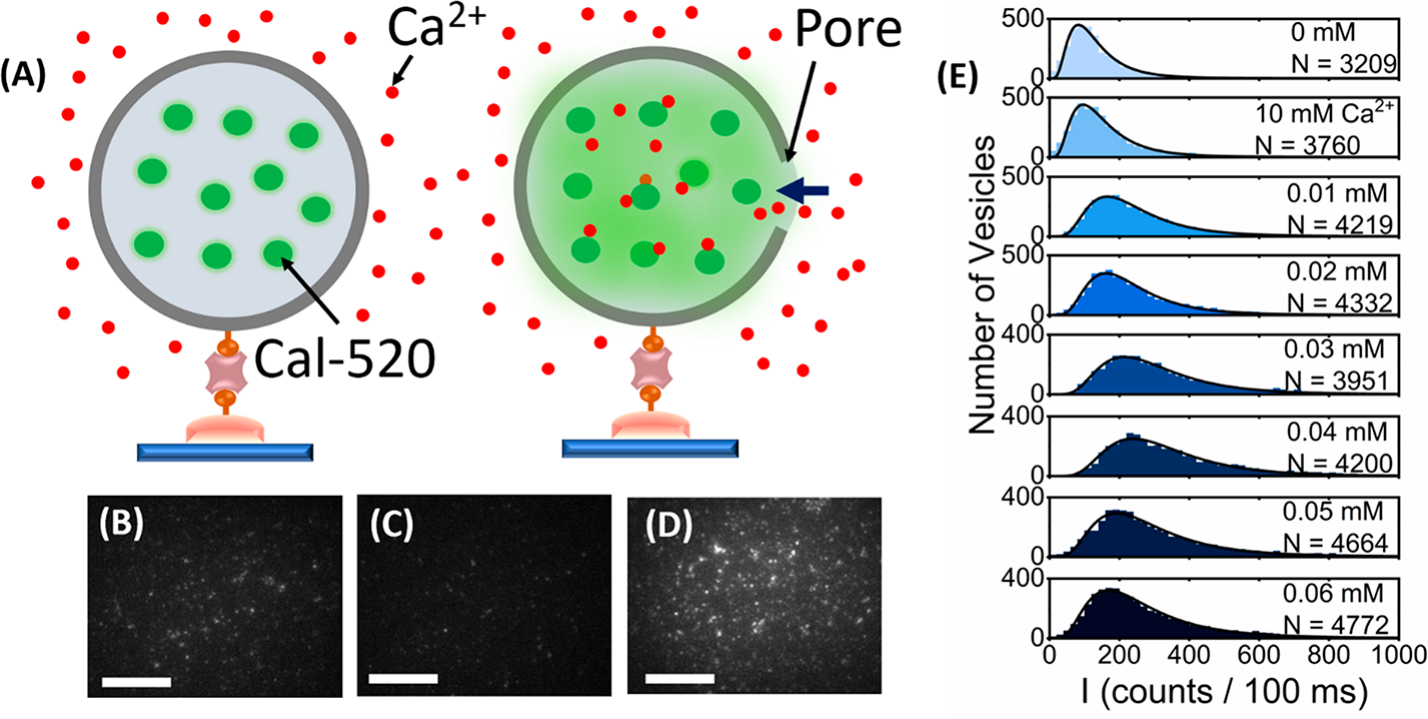
Changes in encapsulated Cal-520 fluorescence intensity upon addition of Tween-20. (A) LUVs encapsulating Cal-520 are placed in Ca^2+^ buffer (left panel). Membrane permeabilization after Tween-20 interaction results in Ca^2+^ influx and Cal-520 intensity enhancement. Wide-field TIRF images of Cal-520-loaded vesicles in (B) imaging buffer (50 mM Tris, pH 8), (C) buffer including 10 mM Ca^2+^, and (D) buffer including 10 mM Ca^2+^ and 0.01 mM Tween-20. (E) Intensity histograms obtained from *N* > 3000 Cal-520-LUVs in imaging buffer (top panel), imaging buffer including 10 mM Ca^2+^, and imaging buffer including 10 mM Ca^2+^ and Tween-20 (lower panels). Solid black lines represent log-normal fits.

A remarkable outcome of this study is the multistep nature of the Tween-20 solubilization mechanism. This involves the deposition of detergent molecules onto the membrane surface, conformational restructuring that can be assigned to expansion, and membrane permeabilization that leads to solution exchange, all prior to lysis. While the current data do not report on the initial dynamics of single surfactants directly interacting with the membrane, and further work in this area would be highly desirable, it is notable that these events cannot be distinguished by using a stand-alone technique, but rather they have emerged by utilizing a multidisciplinary toolkit. Owing to its bulky headgroup and pliable hydrocarbon chain, Tween-20 can be modeled as a cone with positive spontaneous curvature, and thus we attribute the observed structural changes to vesicle instability induced by a mass imbalance between the outer and inner monolayers upon surfactant binding and penetration of the surfactant into the bilayer, the latter of which leads to bilayer bending and the observed swelling behavior.^24,57^ Moreover, LUVs exhibit high radii of curvature which we hypothesize destabilize during the interaction, leading to a reduction in membrane line tension and facilitating the global deformability of the bilayer.^24,58^ This assertion is supported by additional measurements involving the interaction with LUVs incorporating the tension probe FliptR^59^ (Figure S13), whose lifetime depends linearly on membrane tension. Here, a reduction in lifetime from 3.35 ± 0.02 to 2.77 ± 0.03 ns at ∼10× the CMC was observed, indicating probe deplanarization. Even if only a single ion then enters the disrupted vesicle, the local concentration increases by several hundred nanomolar, which is detectable via the Cal-520 assay.^37^ This scenario, while consistent with work suggesting Tween-20 induces bulging of live cells,^4^ differs from those observed by using cholesterol-rich EVs, where no variation in particle size was observed.^50,60^ As previously elaborated, this difference can be qualitatively explained by taking the membrane composition into account, and thus LUVs with no cholesterol content likely facilitate Tween-20 membrane insertion which triggers the observed structural changes. Indeed, a requirement of permeabilization and structural remodeling is membrane insertion, suggesting that Tween-20 may penetrate deeply into the bilayer to trigger the observed effects. This observation of a multistep solubilization mechanism is further supported by previous experiments involving GUVs where Tween-20 gradients induced long-lived pores capable of inducing solution exchange.^58^ A direct comparison between our findings on LUVs and the GUVs used previously is not entirely straightforward due to variations in composition and the fact that optical imaging only provides access to a cross section of the focal plane. In contrast, our approach involves interrogating immobilized vesicles in a microfluidic flow cell where a steady-state detergent concentration can be rapidly reached, and the mean FRET signature arises from dye interactions across the three-dimensional volume of the vesicle. Nevertheless, the vesicle size can be easily tuned, and when 1 μm sized GUVs composed of 99.8% POPC, 0.1% DiI, and 0.1% DiD were studied in the presence of Tween-20, under identical conditions to those shown in Figure 2B, we observed common solubilization attributes: first, an overall reduction in FRET efficiency and, second, an enhancement of the amplitude-weighted average DiI lifetime as the concentration of Tween-20 was progressively increased (Figure S14). An important observation is that the half-maximal concentration constant obtained for GUVs (∼0.13 mM) was double that observed for the LUVs, and the interaction parameter was much smaller (χ ∼ 0.1), indicating that membrane curvature may be a key regulator of the interaction. Overall, GUVs and submicrometer-sized LUVs share similar solubilization attributes including nanoscale partitioning of lipids and the presence of a solution exchange step attributed to permeabilization. Thus, our work on highly curved vesicles is complementary of previous studies and points to common structural remodeling events prior to lysis.

Another aspect of our results that deserves attention is that low concentrations of Tween-20, below the CMC, produced substantial conformational changes. This not only suggests that individual Tween-20 monomers play a role in vesicle swelling and permeabilization but also is particularly striking because nonionic detergents typically achieve membrane solubilization only once above their CMCs. One possible explanation for this is the formation of discrete membrane regions with a high local detergent density that acts as a nucleation site.^61^ This is partially supported by the application of a mass-action model to the ensemble FRET curves, which reveal limited compatibility of the surfactant in the membrane in terms of a Flory–Huggins parameter and the FCS and single-vesicle FRET data which reveal discrete changes to the observed radii. Indeed, our single-vesicle imaging approaches enabled expansion and solution exchange within intact vesicles to be monitored at lipid:detergent ratios of ∼2 × 10^3^, suggesting that structural rearrangements and permeabilization are triggered by <100 Tween-20 monomers per LUV. While the dynamic clustering and insertion of Tween-20 into the bilayer may lead to local invaginations and permeabilization, an alternative explanation for the existence of pores may be related to bilayer bending as the vesicles swell. In all measurements discussed, the composition and curvature change simultaneously, and further work is required to decouple these influences. However, it is worth re-emphasizing that key advantages of the FRET-based approach are that thermodynamic parameters can be assigned to the surfactant–membrane interactions via application of a mass-action model and fluorescently tagged vesicles can be interrogated on a vesicle-by-vesicle basis bypassing major limitations associated with ensemble averaging.

We have established that the combination of ensemble and ultrasensitive single-vesicle spectroscopy approaches can be used to reveal precise molecular level events that underpin Tween-20 induced vesicle solubilization in vitro. Tween-20 dynamically alters the structure and integrity of both freely diffusing and surface-immobilized vesicles via a mechanism involving an initial mass gain, vesicle swelling, membrane permeabilization, and content exchange prior to lysis. Our observations provide new mechanistic insights for how solubilizing detergents perturb and damage highly curved membranes, and may be directly relevant to a number of biotechnological applications where conformational control of the membrane is vital. We also expect that our approaches will find general utility for unmasking vesicle structural changes in response to perturbative agents, including additional surfactants, disruptive proteins, and antiviral agents.

## Supporting Information Available

Experimental methods and materials, QCM-D trajectories, description of the mass-action model, fitting parameters, time-resolved fluorescence decays and lifetimes, size exclusion chromatography data, kinetics, ensemble fluorescence and FRET data, details of the lipid mixing assay, variations in DLS polydispersity index, FCS data and fits, photostability data, FliptR decays, and additional references (PDF)
Transparent Peer Review report available (PDF)
