# Supplementary material for: Tween-20 Induces the Structural Remodeling of Single Lipid Vesicles
Source: J Phys Chem Lett. 2022 Jun 9;13(23):5341–50. doi: 10.1021/acs.jpclett.2c00704 (PMC9208007; doi:10.1021/acs.jpclett.2c00704)
Supplement: Supplementary file 1 — jz2c00704_si_001.pdf [file jz2c00704_si_001.pdf]

Tween-20 Induces the Structural Remodeling of Single Lipid Vesicles.

Lara Dresser[a] , Sarah P. Graham[a], Lisa M. Miller[b], Charley Schaefer[a], Donato Conteduca[a], Steven Johnson[b,d], Mark C. Leake[a,c,d], and Steven D. Quinn[a,d,*]

[a]Department of Physics, University of York, York, UK, YO10 5DD.

[b]Department of Electronic Engineering, University of York, York, UK, YO10 5DD.

[c]Department of Biology, University of York, York, UK, YO10 5DD.

[d]York Biomedical Research Institute, University of York, York, YO10 5DD.

Corresponding Author

*Steven D. Quinn, Department of Physics, University of York, York, UK, YO10 5DD. Email: steven.quinn@york.ac.uk

SUPPORTING INFORMATION

Experimental Methods

Materials. Lipids suspended in chloroform (1-palmitoyl-2-oleoyl-glycero-3-phosphocholine (POPC), 1-oleoyl-2-(12-biotinyl(aminododecanoyl))-sn-glycero-3-phosphoethanolamine (biotin-PE) and Tween-20 were purchased from Sigma Aldrich. Tween-20 was suspended in 50 mM Tris buffer (pH 8) prior to each use. Lipophilic membrane stains (1′-Dioctadecyl-3,3,3′,3′-Tetramethylindocarbocyanine Perchlorate (DiI) and 1,1′-Dioctadecyl-3,3,3′,3′-Tetramethylindodicarbocyanine, 4-Chlorobenzenesulfonate Salt) (DiD)) were purchased from ThermoFisher Scientific. Cal-520, sodium salt was purchased from Stratech and freshly suspended in MilliQ water prior to use. POPC, Biotin-PE and Cal-520 solutions were stored at -20°C, whereas DiI and DiD solutions were stored at 4°C prior to use. All samples were used without additional purification.

Preparation of vesicles incorporating the DiI and DiD FRET sensor. Large unilamellar vesicles composed of 0.1 mol % DiI, 0.1 mol % DiD, 1 % biotin-PE and 98.8 mol % POPC were prepared as previously described[1, 2]. Briefly, lipids, DiI and Did were mixed in chloroform before the solvent was evaporated. The dried lipid film was then resuspended in 50 mM Tris buffer (pH 8) and vortexed. Large unilamellar vesicles were then prepared by the extrusion method, whereby the solutions were extruded at least 21 times through a polycarbonate membrane filter with size cut off of 200 nm. The polydispersity index, as measured by DLS, was 0.478 ± 0.04. On the basis of monodisperse LUVs of 200nm diameter, and reports confirming that both DiI and DiD have excellent membrane-incorporation efficiency and retention, while also minimally perturbing membrane morphology[3], the average number of fluorophores incorporated per vesicle,<N>, was estimated via $\langle N \rangle = \frac{m[D]}{[L]}$, where m is the average number of lipids per vesicle, [D] is the total dye concentration and [L] is the total lipid concentration[4]. The lipid head group was estimated to have a surface area of 0.92 nm^2, resulting in a value of m ~ 2 x 10^5 lipids per vesicle, and $\langle N_{DiI} \rangle = \langle N_{DiD} \rangle \sim 200$. Using a similar approach, POPC vesicles coated in FliptR tension probe were prepared via extrusion using a dye concentration of 4 mM.

Preparation and purification of Cal-520 loaded vesicles. Cal-520 filled vesicles were prepared as previously described[5]. Briefly, POPC and Biotin-PE chloroform stock solutions were mixed such that the POPC: Biotin-PE molar ratio was 100: 1. The chloroform was then removed and the samples were dissolved and vortexed in 50 mM Tris buffer (pH 8) containing 100 mM Cal-520. The solutions were then extruded at least 21 times through a polycarbonate membrane filter with pore size of 200 nm. Vesicle solutions were filtered using a PD-10 desalting column (Sigma Aldrich) and size-exclusion chromatography was performed using an AKTA pure system (GE Healthcare) in order to separate free Cal-520 dye molecules from loaded vesicles.

Quartz Crystal Microbalance with Dissipation (QCM-D) Monitoring. Time-dependent changes in frequency and dissipation were recorded using a Q-Sense E4 (Biolin Scientific) system. SiO_2-coated sensors (Biolin Scientific) with a fundamental frequency of 5 MHz were treated with UV/ Ozone for 10 minutes, then sonicated in 2

% Hellamanex III for 10 minutes and ultrapure Milli-Q water for 20 minutes and dried under N_2 flow. The sensors were then treated with UV/ Ozone for a further 30 minutes, immersed in ethanol and dried with N_2 flow prior to use. Each sensor was pre-functionalized with an amine monolayer by immersion in 4% v/v ATES/IPA solution for 16 hours, followed by washing with IPA and dried with N_2 before installation in the flow modules. The sensors were then flushed with 50 mM Tris buffer (pH 8) at 20 mL / min until a stable baseline was reached. At the start of each experiment, the sensor surfaces were coated with 0.1 mg/mL biotinylated BSA (Sigma Aldrich) and 1 mg/mL BSA (Sigma Aldrich) dissolved in 50 mM Tris buffer (pH 8). When saturation was reached, the sensors were rinsed with buffer to remove unbound molecules, before NeutrAvidin (ThermoFisher Scientific) was added at 0.2 mg/mL in 50 mM Tris buffer (pH 8) until saturation was reached and a further rinse step with buffer was performed. Vesicles containing 1 mol % biotin-PE and 99 % POPC were then added to the surface using a final lipid concentration of 25 mg/mL in 50 mM Tris buffer until saturation was reached. This was followed by a further rinse step and the insertion of Tween-20 detergent solutions in 50 mM Tris buffer (pH 8) at the specified concentrations.

Steady State Fluorescence and FRET Spectroscopy. Fluorescence emission spectra were measured using continuous wave excitation under magic angle conditions with a HORIBA Fluoromax-4 spectrophotometer. Spectra from vesicles containing DiI and DiD were recorded using an excitation wavelength of 520 nm, whereas an excitation wavelength of 493 nm was used for measuring Cal-520 filled vesicles. Apparent FRET efficiencies, E, were determined via $E = I_A/(I_A+I_D)$ where I_D and I_A represent the fluorescence emission intensities of DiI and DiD, respectively. FRET efficiency plots were fitted using $E = A + B\phi$, where A and B are the measured FRET efficiencies at 0 mM Tween-20 and at the end of the titration, respectively, and$0 \leqslant \phi \leqslant 1$is the volume fraction of lipids in the membrane. As molecular models, we used the Hill model, $\phi = 1/(1 + ([Tween - 20]/k)^n)$, with k the half-maximal concentration and n the Hill coefficient, and we used a mass-action model for mixed micellisation $Kexp(-\phi^2\chi)([Tween - 20]/(1-\phi) - [lipid]/\phi) = 1$, where K is an equilibrium constant and χ is the Flory-Huggins interaction parameter (Supplementary Text 1). The relative influx, φ, of calcium into vesicles upon o addition of Tween-20 was determined via $\varphi = \frac{I_v - I_b}{I_I - I_b}$ where I_v is the Cal-520 intensity after addition of Tween-20, I_b is the Cal-520 intensity in the absence of Tween-20 and I_I is Cal-520 intensity after addition of 1mg/mL ionomycin[5, 6]. The data points shown in Figure 2 represent the mean and standard error of the mean from 3 individual experimental runs and all experiments were performed in 50 mM Tris buffer (pH 8) with a final POPC concentration of 25 mM.

Fluorescence Lifetime Measurements. Time-resolved fluorescence spectroscopy on DiI and DiD loaded vesicles was performed using a FluoTime300 time-correlated single photon counting spectrophotometer equipped with a hybrid PMT detector (PMYA Hybrid 07, Picoquant). Time-resolved fluorescence decays were measured under magic angle conditions using pulsed excitation at 532 nm with a repetition rate of 50 MHz (LDH-D-FA-530L, Picoquant). Fluorescence emission decays at 565 nm,

corresponding to the peak of DiI emission, were collected until 10^4 counts accumulated at the decay maximum. Fluorescence decay curves were fitted by iterative re-convolution of the instrument response function and the observed fluorescence decay using a multi-exponential decay function of the form $I_t = \sum_{i=1}^{n} a_i e^{\frac{-t}{\tau_i}}$ where I_t is the intensity at time, t, normalized to the intensity at t = 0, and t_i and a_i represent the fluorescence lifetime and fractional amplitude of the i'th decay component. The quality of the fit was judged based on the convergence of the reduced chi-squared. All experiments were performed in 50 mM Tris buffer (pH 8) with a final POPC concentration of 25 mM. In the context of FliptR measurements, fluorescence lifetime decays were acquired at 600 nm using a pulsed 485 nm laser (LDH-P-C-485, Picoquant) with a repetition rate of 20 MHz.

Dynamic Light Scattering. Vesicle size distributions in the absence and presence of Tween-20 were measured using a Zetasizer mV DLS system (Malvern Instruments). A final POPC concentration of 50 mM in 50 mM Tris buffer (pH 8) was used in all experiments. Briefly, the Brownian motion of vesicles in suspension scattered laser light at 632.8 nm, and correlation of the intensity fluctuations yields the diffusion coefficient and hydrodynamic radius via the Stokes-Einstein relationship as previously described[7, 8]. All DLS data were collected using 178° backward scattering and averaged over three experimental runs. The refractive index of the dispersion medium was 1.33. All sizes discussed are in terms of spherical hydrodynamic radii, and all DLS analyses are reported in terms of intensity distribution.

Fluorescence Correlation Spectroscopy. Fluorescence correlation spectroscopy (FCS) measurements were performed on a Zeiss LSM 880 microscope, using a GaAsP detector as previously described[7]. Samples composed of 99.9 mol % POPC and 0.1 mol % DiI were pipetted onto microscope slides, sealed with silica and a 1.5 coverslip. Samples were excited with a 514 nm excitation line with typical powers of 4 mW as measured at the sample plane. Final concentrations of POPC and DiI in 50 mM Tris buffer (pH 8) were 70 mM and 80 nM, respectively. The confocal volume was measured using a calibration sample of 6 nM Rhodamine-6G at 21°C and constraining the diffusion coefficient to be ~400 mm^2s^{-1}. Autocorrelation curves, G(t) were fitted to an expression of the form $G(\tau) = y_0 + \frac{1}{n}\left(1 + \frac{\tau}{\tau_{diff}}\right)\sqrt{\frac{1+\tau}{SP^2\tau_{diff}}}\left(1 + \frac{T}{1-T}\right)e^{\frac{-\tau}{\tau_{trip}}}$ where y_0, n, τ, τ_{diff}, SP, T and τ_{trip} are offset, effective number of particles in confocal volume, lag time, residence time in confocal volume, structure parameter, fraction of particles in triplet state and residence time in triplet state respectively. Diffusion coefficients, D, were then determined by $D = \frac{r_0^2}{4\tau_D}$, where r_o is the spot width (~0.238 mm).

Single Vesicle FRET Spectroscopy. Microfluidic flow cells were constructed as described previously[9] and coated with 0.1 mg/mL BSA-Biotin, 1 mg/mL BSA and 0.2 mg/mL Neutravdin as described for our QCM-D monitoring approach. Biotinylated POPC vesicles containing 0.1 mol % DiI and 0.1 mol% DiD were then added to a final concentration of 70 mg/mL in imaging buffer (50 mM Tris, 6 % (W/V) D-(+)-glucose

containing 1 mM Trolox and 6.25 mM glucose oxidase and 0.2 mM catalase) and incubated for 15 minutes at room temperature to achieve a surface coverage of ~150-200 vesicles per 50 x 50 mm field of view. Unbound vesicles were then removed by washing the flowcell with imaging buffer. Bespoke TIRF microscopy was then performed on an inverted microscope (Nikon Eclipse Ti) containing a CFI Apo TIRF 100 x NA 1.49 oil-immersion objective lens (Nikon) and illumination from a TEM_{00} 532 nm line (Obis, Coherent). Emission was separated from the excitation line via a dichroic and emission filter mounted beneath the lens turret (Chroma 59907-ET-532/640). DiI and DiD emission was then spatially separated using a DualView image splitter containing a dichroic filter (T640LPXR, Chroma) and band pass filters (ET585/65M and ET700/75M, Chroma) and imaged in parallel on a back-illuminated Prime 95b CMOS camera cooled to -30ºC (Photometrics). After each addition of Tween-20 in imaging buffer, movies were acquired with 50 ms exposure time. Recorded images were then analysed in MATLAB (R2019a) using iSMS single-molecule FRET microscopy software[8]. Briefly, the donor and acceptor emission channels were aligned, and background-corrected DiI and DiD emission trajectories were obtained by integration of the intensity within the area containing the vesicle signal for each time point. Apparent FRET efficiencies were calculated as described previously, and related to the mean distance between probes, R, via $E = \frac{R_0^6}{R_0^6+R^6}$, where R_o = 5.3 nm is the Förster radius.
Scanning Electron Microscopy

Scanning electron microscopy. SEM was performed using a JEOL JSM 7800-F system operating at 5kV. Vesicles were prepared in 50 mM Tris (pH 8) containing Tween-20 at the specified concentrations, diluted ~10 x in deionized water and vortexed. A 30 mL volume of the vesicle solution was then added to a silicon substrate and the solution evaporated. The substrate was then covered with a 5 nm Pt/Pd layer to avoid any charging effects or disruption of the vesicles in the microscope. Vesicle diameters were then determined using ImageJ, where automated analysis of black-and-white binary images enabled separation of regions of white pixels (the vesicles) against a dark background. Vesicle circularity was measured via $4p(A/p^2)$, where A is the observed area and p is the perimeter. In this case, a circularity value of 1 indicates a perfect circle, whereas a circularity value approaching 0 indicates an increasingly elongated polygon.

Single Vesicle Measurements of Ca^{2+} Influx. Biotinylated POPC vesicles encapsulating Cal-520 at a concentration of 200 mM were prepared and immobilized as previously described. Tethered vesicles were then incubated with imaging buffer containing 10 mM Ca^{2+} and imaged via TIRF using 488 nm excitation. Cal-520 fluorescence was separated from incident excitation using a dichroic mirror of edge wavelength 495 nm and emission bandpass filter (502-548 nm) and imaged using objective-based TIRF. Next, Ca^{2+} rich imaging buffer containing Tween-20 at concentrations specified in the main text were added to the flowcells and images of Ca^{2+} saturated vesicles were acquired with an exposure time of 100 ms.

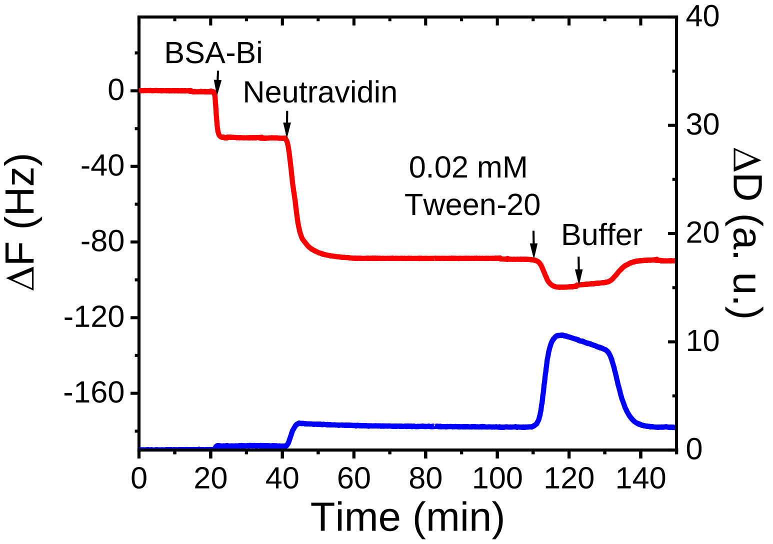

Figure S1. The non-specific and reversible attachment of Tween-20 to a SiO_2 sensor surface containing BSA, BSA-Biotin and Neutravidin. The time evolution of ΔF (red) and ΔD (blue) upon the addition of BSA-Biotin, Neutravidin and 0.02 mM Tween-20 to a SiO_2 sensor surface at 21°C. After saturation of the surface by Tween-20 was reached at t = 120 min, the sensor was flushed with 50 mM Tris buffer (pH 8).

Supplementary Text 1: Mass-Action Model for Mixed Micellization.

The ensemble FRET efficiency of POPC vesicles is interpreted to be proportional to the surface concentration of interacting DiI and DiD in the membrane, [DiI*DiD]=K[DiI][DiD] relative to the maximum surface concentration of interaction pairs, min([DiI], [DiD]). In our experiments both concentrations are equal, [Di]=[DiI]=[DiD], so $E_{FRET} \propto [Di]$. A decrease in the FRET efficiency occurs if this surface concentration decreases, which occurs upon swelling of the vesicles when Tween-20 molecules are incorporated in the membrane. Hence, we may write

$$E_{FRET} = A + B\phi, \qquad \text{(Equation 1)}$$

where *A* and *B* are constants, ϕ is the volume fraction of lipids, and 1-ϕ is the volume fraction of Tween-20 in the membrane (assuming the concentration of FRET tags is very low). In this section we focus on the molecular mechanism by which ϕ depends on the experimentally tunable overall concentration of Tween-20.

In previous work[1], we heuristically applied the Hill model to describe the self-assembly curves, ϕ=1/(1+([Tween-20]/k)^n), where *k* is the half maximal concentration and *n* is the Hill coefficient, which controls how sharp the self-assembly transition is at high concentrations. This approach has been useful to characterise a solubilization concentration (in terms of the half maximal concentration) and the sharpness of the transition, but gives a poor fit quality at low concentrations below the transition, which indicates there are some pieces of physics missing. Indeed, the Hill model assumes that groups of *n* molecules cooperatively adsorb to a limited number of binding sites at the vesicle without taking into account that 1. the molecules may adsorb independently, 2. the adsorbed molecules have some translational entropy in the membrane and exhibit interactions with other adsorbed molecules and the lipids, 3. the membrane does not have a limited number of binding sites and may in principle swell indefinitely. Hence, in the present work we develop a simple mass-action model for mixed micellization that takes this physics into account[8].

As a starting point, we consider a solution in a volume *V* at temperature *T* with a total number density of lipids, ρ_L (we presume all lipids to reside in the membrane), and a total number density Tween-20, $\rho_T = N_A[Tween-20]$(with N_A Avogadro's constant), which is partitioned into a concentration that is free in solution, $\rho_{T,s}$, and a concentration that is adsorbed to the vesicle $\rho_{T,v}$, hence, $\rho_T = \rho_{T,s} + \rho_{T,v}$, and the volume fraction of lipids in the membrane is $\phi = \rho_{L,v}/(\rho_{T,v} + \rho_L)$. We will now formulate the free-energy contributions of the lipids and of freely dissolved and adsorbed Tween-20, and minimise the overall free energy to obtain the dependence of ϕ on $\rho_T$via $\rho_{T,v}$.

A Tween-20 molecule that is free in solution has translational entropy, which provides a free energy contribution $kTln(\rho_{T,v}V_m) - kT$, with *k* Boltzmann's constant and with V_m the molecular volume. To adsorb to the vesicle, it loses this translational entropy, but gains a binding enthalpy *h*<0, as well as translational entropy within in the membrane. If self-interactions are preferred over interactions with the lipids, this repulsive interaction is captured using the mean-field interaction energy $\phi(1-\phi)\chi kT$ per molecule, with χ the Flory-Huggins parameter. This free energy of mixing,

$$f_{mix}(\phi) = [\phi ln\phi + (1-\phi)ln(1-\phi)]kT + \phi(1-\phi)\chi kT \qquad \text{(Equation 2)}$$

is contributed to by both the Tween-20 molecules and the lipids in the membrane. Finally, we take into account that each Tween-20 molecule has a chemical potential *μ*, which acts as a Lagrange multiplier, as it enables us to minimise the total free energy density

$$\frac{F}{V} = \rho_{T,s}\left[kTln\left(\rho_{T,s}V_m\right) - kT - \mu\right] + \rho_{T,v}[f_{mix}(\phi) + h - \mu] + \rho_L f_{mix}(\phi) \qquad \text{(Equation 3)}$$

with respect to $\rho_{T,s}$ and $\rho_{T,v}$independently, and the constraint $\rho = \rho_{T,s} + \rho_{T,v}$ is obeyed by adjusting *μ* accordingly. Indeed, by minimising the free energy density with respect to $\rho_{T,s}$ we obtain $\mu = kTln\left(\rho_{T,s}V_m\right)$, and minimization with respect to $\rho_{T,v}$ gives $0 = f_{mix} + h + \left(\rho_{T,v} + \rho_L\right)\frac{df_{mix}}{d\rho_{T,v}} - \mu$. After substituting the chemical potential, and using the product rule, $df_{mix}/d\rho_{T,v} = (df_{mix}/d\phi)\left(d\,\phi/d\,\rho_{T,v}\right)$, this gives $0 = ln(1-\phi) + \phi^2\chi + h/kT - ln\left(\rho_{T,s}V_m\right)$. We now introduce the equilibrium constant for binding, K=V_m exp(–h/kT)≡V_m exp(–$\Delta H/RT$), with ΔH the binding enthalpy and $R=kN_A$ the gas constant, and substitute $\rho_{T,s} = \rho_T - \rho_L\,(1-\phi)/\phi$, to obtain the final result

$$Kexp(-\phi^2\chi)\left(\frac{\rho_T}{1-\phi} - \frac{\rho_L}{\phi}\right) = 1 \qquad \text{(Equation 4)}$$

which is an implicit equation for ϕ as a function of $\rho_T = N_A[Tween - 20]$, with as parameters the equilibrium constant *K*, interaction parameter χ, and the overall lipid concentration ρ_L.

To inspect how these parameters affect the titration curve, we first focus on the asymptotic limit where Tween-20 and the lipid are perfectly miscible in the membrane, i.e., we consider the case χ=0. Here, the volume fraction of lipids in the membrane is given by

$$\phi = \frac{1}{2}\left(1 - K(\rho_T + \rho_L) + \sqrt{(1 - K(\rho_T + \rho_L)^2 + 4K\rho_L)}\right) \qquad \text{(Equation 5)}$$

In **Figure S2** we plot this curve for various lipid concentrations ρ_L. We find that this concentration shifts the half-maximal concentration as $\rho_{1/2} = \rho_L + 1/(2K)$ (Hence, if the data would be analysed using the Hill model, this would yield $k \approx (1 + 2\rho_L K)/2\,K$). Further, we find that the transition is broad and smooth at high lipid concentrations, but that the transition becomes sharp at $K\rho_T = 1$ for vanishing lipid concentrations. Within the Hill model, this behaviour would be attributed to an increasing cooperativity of Tween-20 adsorption; our new interpretation is that a sharper transition indicates a larger excess of Tween-20 relative to the number of lipid molecules at the transition.

The influence of the interaction parameter can be assessed in the regime where it remains small, χ<<1. Here, $exp(-(1-\phi)^2\chi) \approx -(1-\phi)^2\chi$ can be inserted, which yields the cubic equation $K(\rho_T + \rho_L)\phi^3 - (1 + K\rho_L)\phi^2 + \left(1 - K(\rho_T + \rho_L)\right)\phi + K\rho_L = 0$. For low lipid concentrations, again the transition becomes sharp (ϕ=0 for $K\rho_T > 1$), and below the transition concentration, $K\rho_T < 1$, we have

$$\phi = \frac{1}{2\chi K\rho_T}\left(1-\sqrt{1-4\chi K\rho_T(1-K\rho_T)}\right), \quad \text{(Equation 6)}$$

which we plot in **Figure S2**. For low concentrations, $K\rho_T \ll 1$, this gives $\phi = 1-(1-\chi)K\rho_T$, and resembles the non-cooperative Hill model (i.e., for *n*=1) if χ=0. Hence, from the shape of the curve we assess information about the interaction of Tween-20 with the lipids.

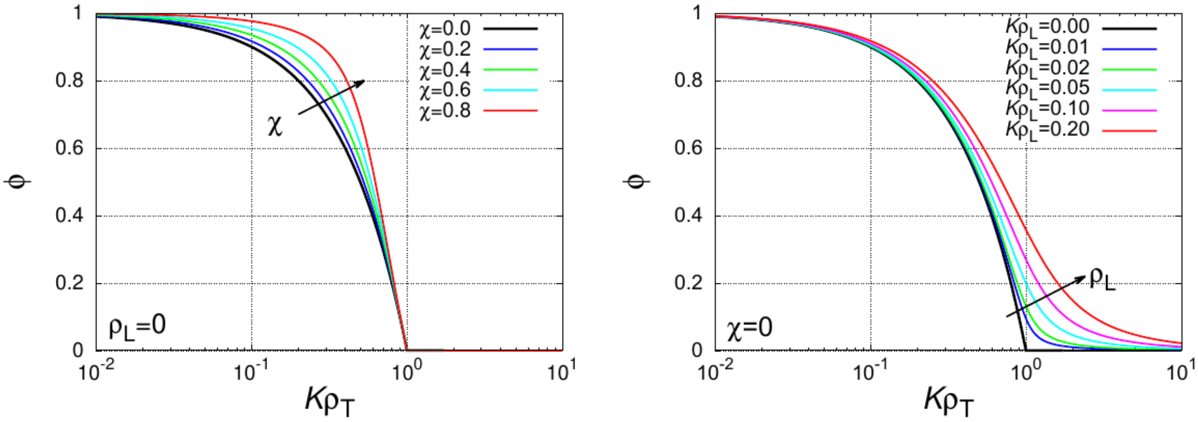

Figure S2. Adsorption isotherm of Tween-20 to a vesicle. The volume fraction of lipids in a membrane, ϕ, against the Tween-20 concentration, ρ_T. The curves are controlled by the equilibrium constant *K*, the concentration of lipids, ρ_L, and by the interaction parameter χ. For low lipid concentrations, $\rho_L \to 0$, there is a sharp transition, and the shape of the curve is controlled by χ. For ideal mixing of Tween-20 with the lipids, χ=0, the transition becomes less sharp with an increasing lipid concentration.

We fit both the Hill model and the mass-action model to our E_{FRET} data to assess the fit quality and extract the physical parameter values. First, we determine the baseline value *A* in Eq. (1) at high concentrations. From this we obtain the experimental variance σ_E^2. We then use this to calculate the reduced chi square definition

$$\chi^2 = \frac{1}{N_{data}-N_{par}} \sum_{i=1}^{N_{data}} \frac{\left(E_{i,fit}-E_{i,data}\right)^2}{\sigma_E^2} \quad \text{(Equation 7)}$$

where N_{par}=3 for the Hill model (which has parameters *B*, *k*, and *n*) and N_{par}=4 for the mass-action model (which has parameters *B*, *K*, χ, ρ_L). We note that ρ_L is the concentration of lipids, which is strictly known. Using the Van ‘t Hoff relationship $K \propto exp(\Delta H/RT)$for the equilibrium constant K, we found an enthalpy *ΔH*=– 31 +/- 3 kJ/mol for binding surfactants to a lipid membrane.

Table S1. Fitting parameters associated with mass action model fits applied to the E_{FRET} titrations shown in Figure 2B.

	**4 °C**	**21 °C**	**37 °C**
A	0.055 ± 0.002	0.55 ± 0.002	0.55 ± 0.002
B	0.382 ± 0.007	0.382 ± 0.007	0.382 ± 0.007
ρ_L (mM)	0.0022 ± 0.0004	0.0022 ± 0.0004	0.0022 ± 0.0004
K (mM^{-1})	6.27 ± 0.11	11.93 ± 0.32	26.39 ± 1.06
χ	1.15 ± 0.12	1.22 ± 0.17	1.24 ± 0.21

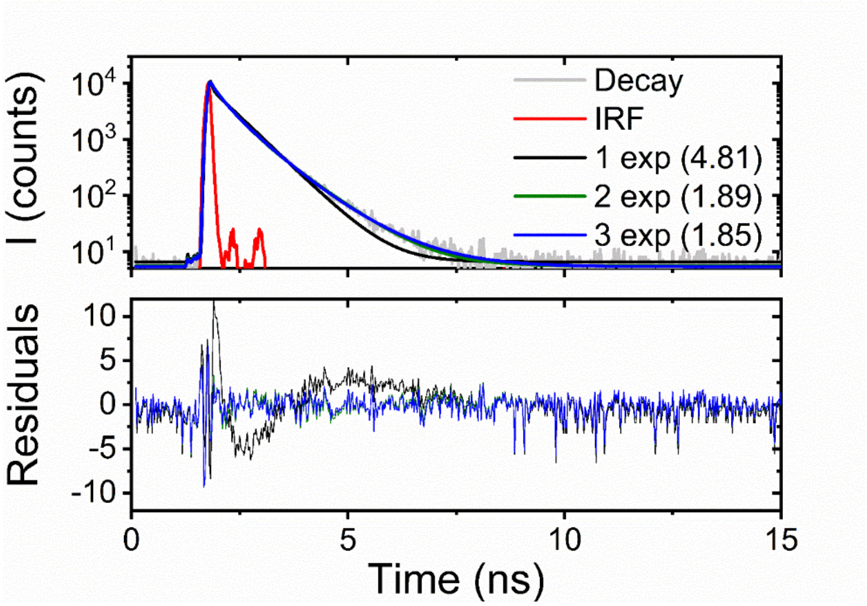

Figure S3. Time-resolved fluorescence decays obtained from DiI and DiD loaded vesicles fit to a tri-exponential model. Top panel: Representative time-resolved fluorescence decay (grey) obtained from DiI and DiD coated POPC vesicles in the absence of Tween-20. Black, green and blue solid lines represent reconvolution fits to mono-, bi- and tri-exponential decay functions, respectively. The red solid line represents the instrument response function (IRF). λ_{ex} = 532 nm (50 MHz). The numbers in brackets represent goodness of fit, χ^2, values. Bottom panel: residuals obtained for the mono-, bi- and tri-exponential decay functions. Solution conditions:25 μM POPC, 0.025 μM DiI, 0.025 μM DiD, 50 mM Tris, pH 8.0.

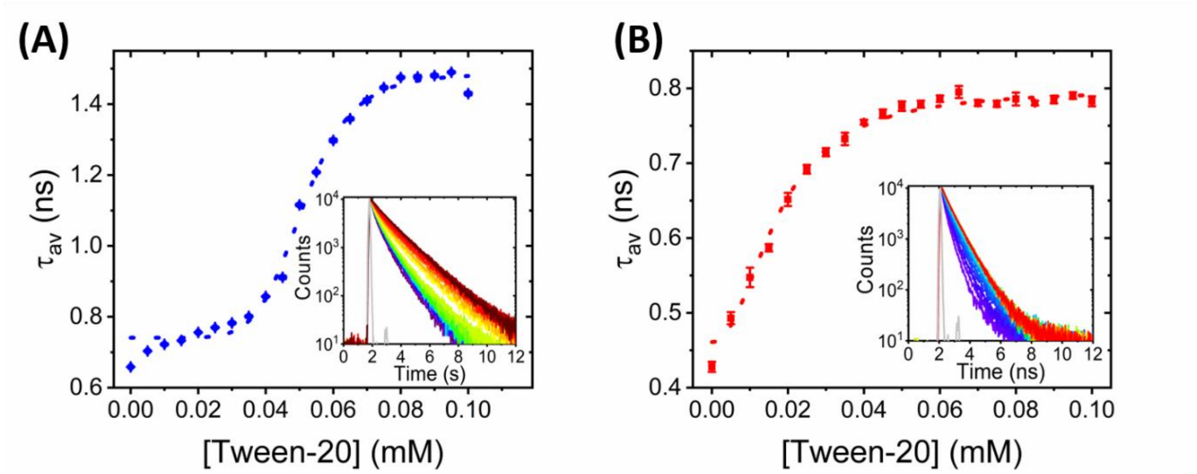

Figure S4. Time-resolved fluorescence decays obtained from DiI and DiD loaded vesicles as a function of Tween-20 at 4ºC and 37ºC. The amplitude weighted average lifetime of DiI in the presence of DiD as a function of Tween-20 at (A) 4ºC and (B) 37ºC with λ_{ex} = 532 nm (50 MHz). Insets: the corresponding time-resolved fluorescence decays. The instrumental response function acquired under both conditions is shown in grey. Solution conditions: 25 μM POPC, 0.025 μM DiI, 0.025 μM DiD, 50 mM Tris, pH 8.0.

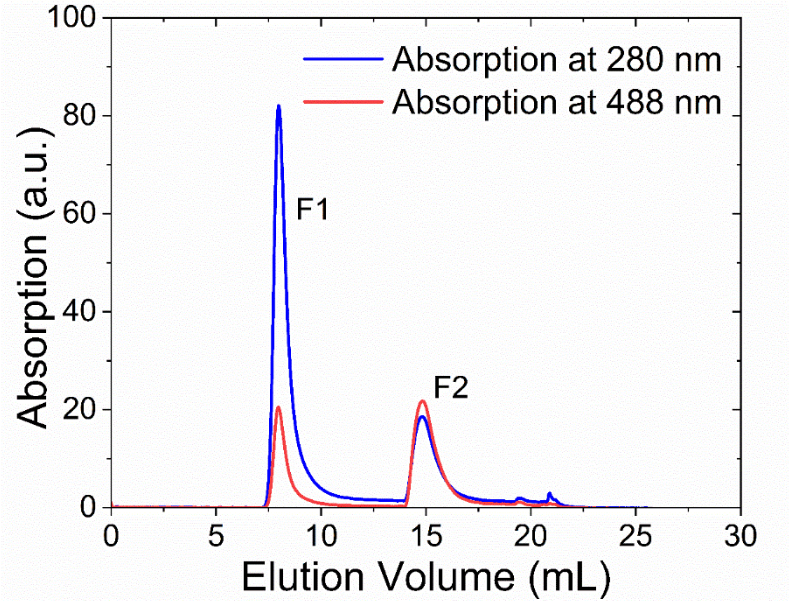

Figure S5. Separation of Cal-520 loaded POPC vesicles from non-incorporated Cal-520 via size exclusion chromatography. 200 nm sized POPC vesicles were prepared in 50 mM Tris buffer (pH 8.0) with 100 mM Cal-520 by the extrusion method. Size-exclusion chromatography was then performed using a 10/30 column attached to an AKTA pure system (GE Healthcare). Absorption was monitored at 280 nm (blue) and 488 nm (red). Fractions F1 and F2 represent the vesicle fraction and free dye/lipid fraction respectively.

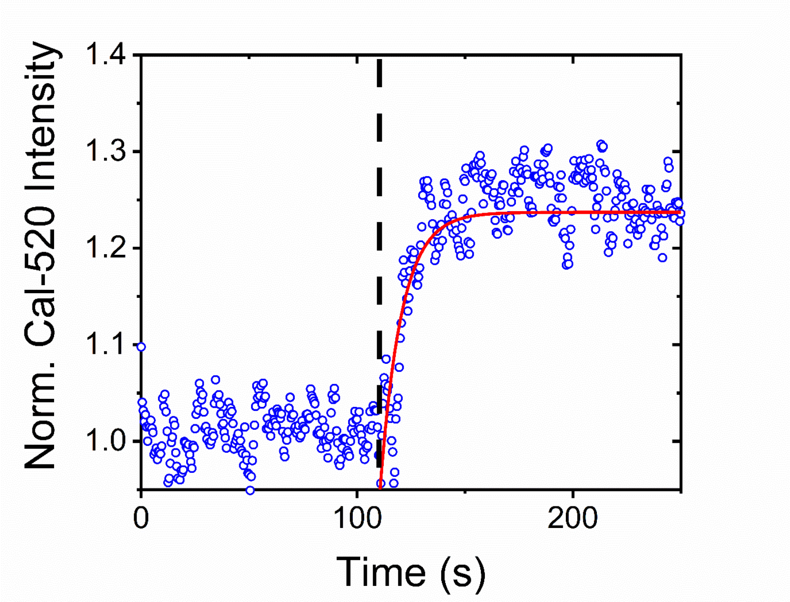

Figure S6. Kinetics of Tween-20 induced membrane disruption followed by the fluorescent enhancement of Cal-520. Normalized variation in the fluorescence emission intensity obtained from POPC vesicles encapsulating Cal-520 before (< 120 s) and after (> 120 s) injection of 0.2 mM Tween-20. The dashed line represents the point of Tween-20 injection and the solid red line represents a mono-exponential fit.

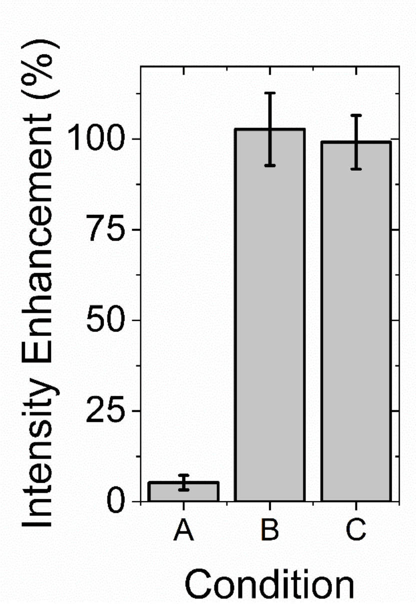

Figure S7. Comparative bar plot summarizing the relative variation in fluorescence intensity enhancement observed from Cal-520 loaded POPC vesicles in (A) 50 mM Tris (pH 8), 1 mM Ca^{2+}, (B) 50 mM Tris (pH 8), 1 mM Ca^{2+}, 1 mg/mL ionomycin and (C) 50 mM Tris (pH 8), 1 mM Ca^{2+}, 0.06 mM Tween-20. Error bars represent the standard error of the mean from 3 experimental runs. Solution conditions: 25 μM POPC, 1 μM Cal-520.

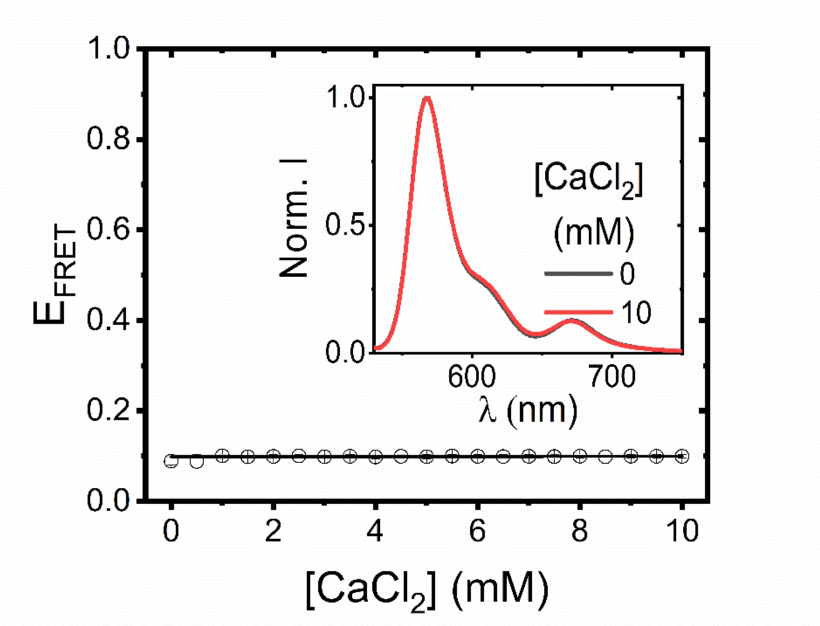

Figure S8. Ca^{2+} does not induce POPC vesicle fusion. Representative variation in E_{FRET} associated with two populations of POPC LUVs (one containing 1 % DiI (donor) and the other containing 1 % DiD (acceptor)) at a 1: 1 ratio in 50 mM Tris buffer (pH 8) in the absence and presence of $CaCl_2$. Inset: the corresponding normalized variation in fluorescence emission spectra (λ_{ex} = 520 nm) in the absence (black) and presence (red) of 10 mM $CaCl_2$.

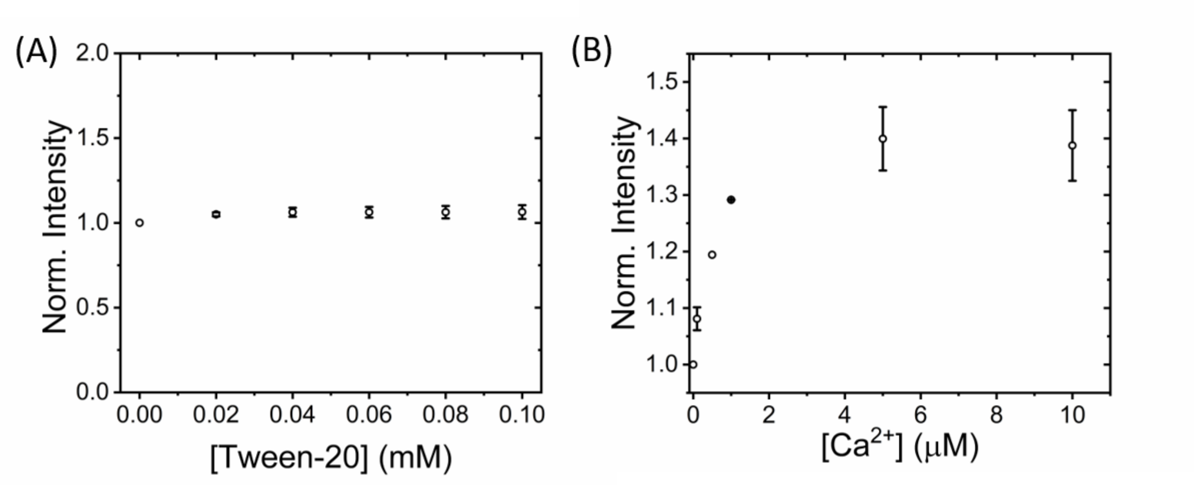

Figure S9. Representative variation in free Cal-520 emission intensity. Normalized variation in the fluorescence emission intensity of 0.1 μM Cal-520 as (A) a function of Tween-20 and (B) as a function of Ca^{2+} in the presence of 0.2 mM Tween-20. In both cases, the base buffer was 50 mM Tris, pH 8.0, 21°C.

Table S2. Fitting parameters and errors associated with Hill Model fits applied to the Cal-520-loaded vesicle titrations shown in Figure 2(D).

	**4 °C**	**21 °C**	**37 °C**
A	1.04 ± 0.02	1.04 ± 0.06	1.03 ± 0.08
B	1.92 ± 0.03	1.84 ± 0.02	1.93 ± 0.04
k (mM)	0.05 ± 0.01	0.015 ± 0.01	0.022 ± 0.003
N	2.75 ± 0.28	2.53 ± 0.43	1.90 ± 0.39
R^2	0.99	0.98	0.97

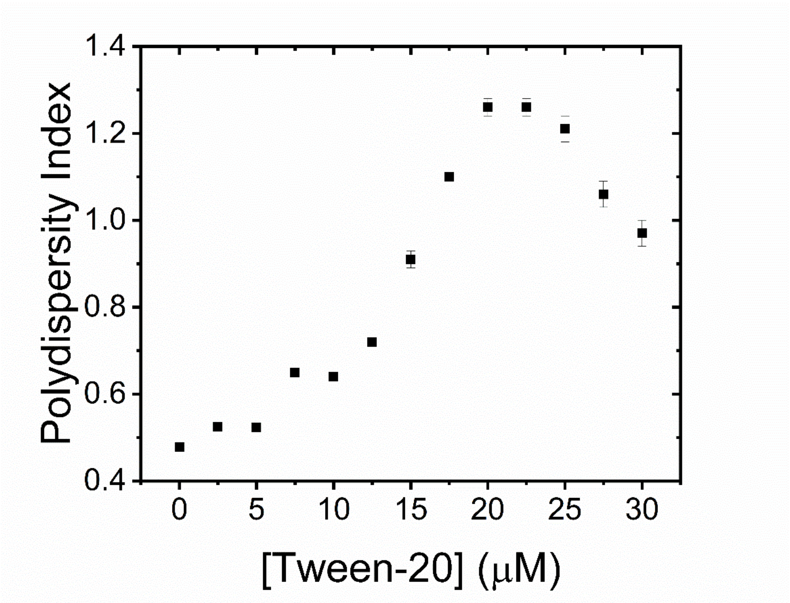

Figure S10. Polydispersity index of POPC vesicles in the presence of Tween-20. Variation in the polydispersity index of POPC vesicles suspended in 50 mM Tris (pH 8.0) buffer in the absence and presence of Tween-20. Data points represent the mean (± SEM) from 3 separate experimental runs. Solution conditions: 25 μM POPC, 0.025 μM DiI, 0.025 μM DiD, 50 mM Tris, pH 8.0.

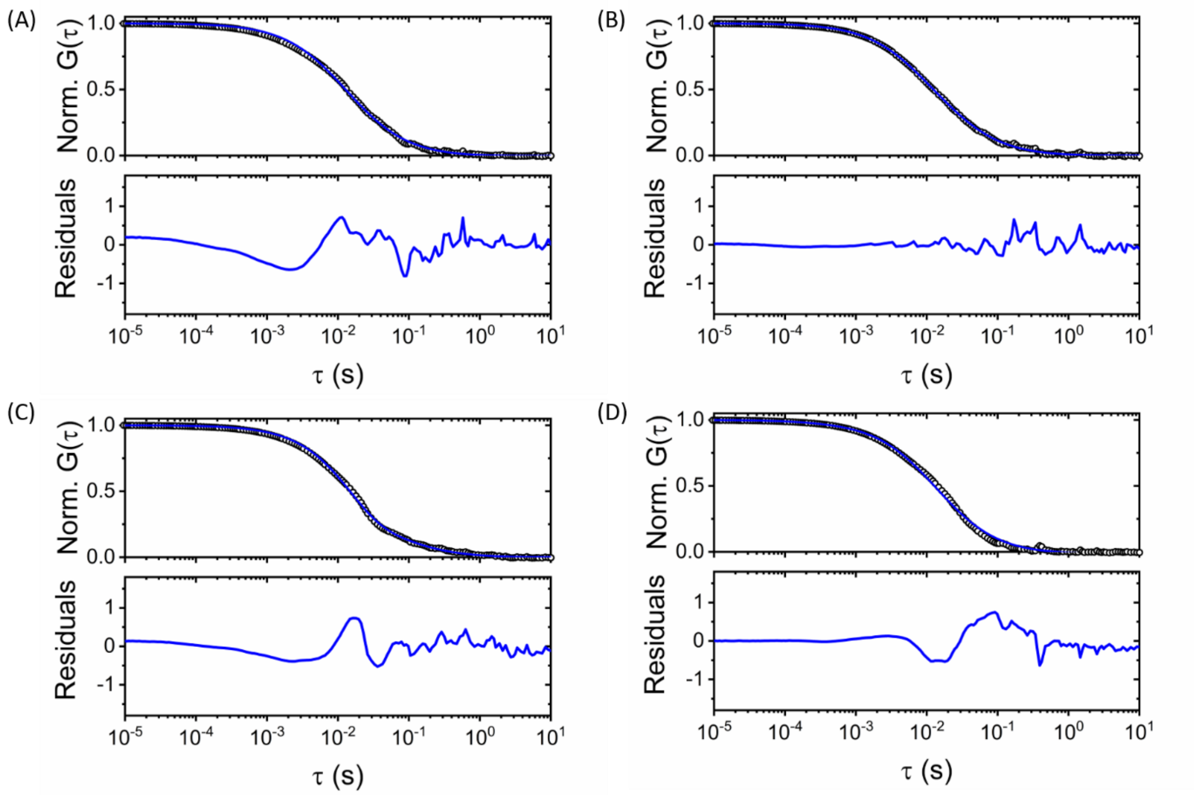

Figure S11. Tween-20 vesicle interactions reported using FCS. Cross correlation curves (black) and fits (blue) (top panel) associated with 200 nm sized POPC vesicles containing 0.1 % DiI in the presence of (A) 0 mM, (B) 0.02 mM, (C) 0.04 mM and (D) 0.06 mM Tween-20. Bottom panels represent residuals of the fits.

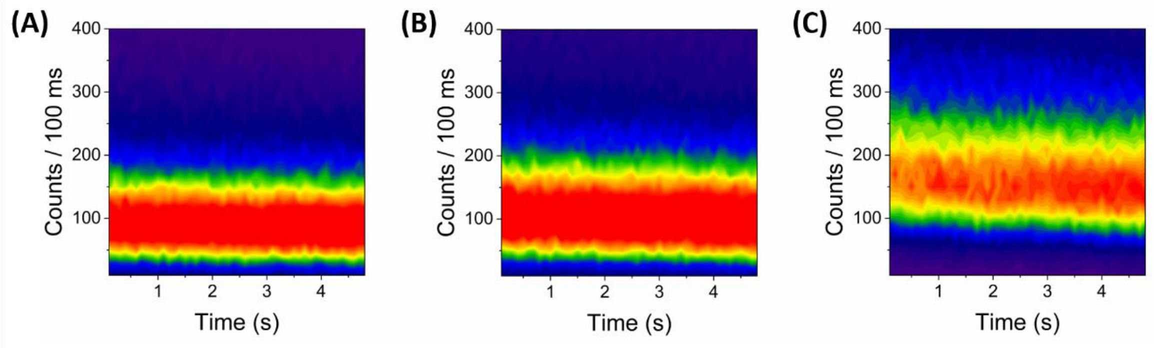

Figure S12. Photostability of Cal-520 loaded vesicles in the absence and presence of Tween-20. Contour plots of the time-evolution of Cal-520 vesicle population in (A) 50 mM Tris buffer, pH 8, (B) 50 mM Tris buffer, 10 mM Ca^{2+}, pH 8 and (C) 50 mM Tris buffer, 10 mM Ca^{2+}, 0.01 mM Tween-20, pH 8. Each contour plot was produced by superimposing individual intensity trajectories and contours are plotted from blue (lowest population) to red (highest population).

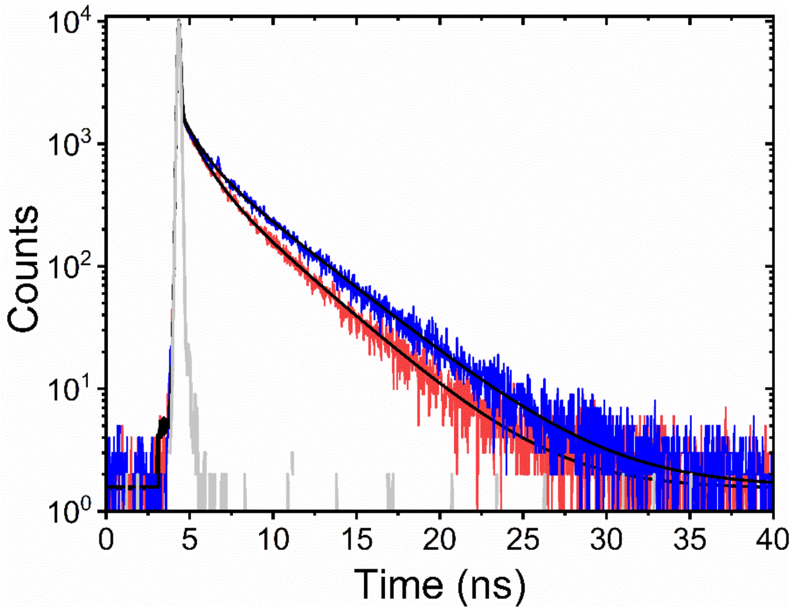

Figure S13. Reduction in membrane tension monitored by FliptR response. Time-resolved fluorescence decays obtained from POPC vesicles coated in FliptR in the absence (blue) and presence of 0.5 mM Tween-20 (red). Solid black lines represent tri-exponential fits to the experimental data. Also shown is the instrumental response function (grey). Solution conditions: 25 μM POPC, 0.4 μM FliptR, 50 mM Tris, pH 8.

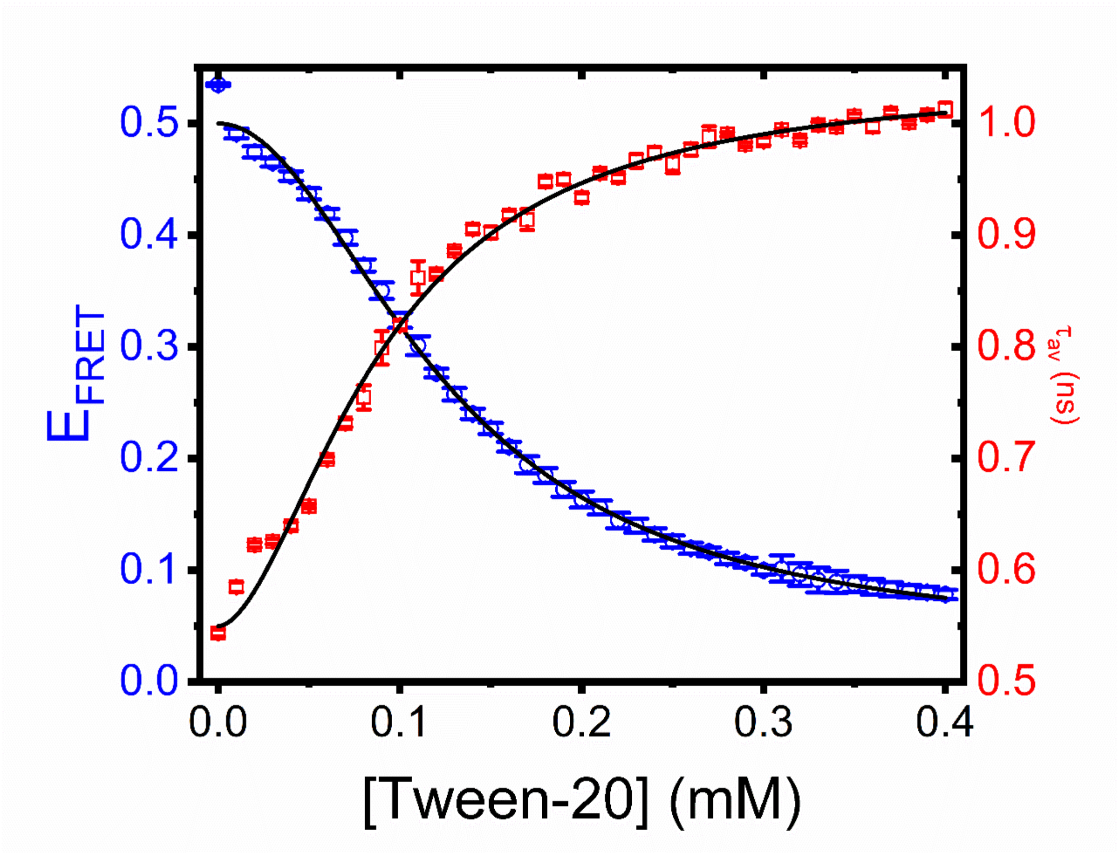

Figure S14. Interaction between Tween-20 and 1 μm sized GUVs monitored by ensemble FRET spectroscopy and time-correlated single photon counting. Variation in the ensemble FRET (blue) efficiency of 1 μm sized GUVs composed of 99.8 % POPC, 0.1 % DiI and 0.1 % DiD as a function of Tween-20 concentration. Also shown is the representative variation in DiI lifetime across the titration (red). Solid black lines correspond to Hill Model fits. Solution conditions: 25 μM POPC, 0.025 μM DiI, 0.025 μM DiD, 50 mM Tris, pH 8, 21°C.

References

(1) Dalgarno, P. A.; Juan-Colas, J.; Hedley, G. J.; Pineiro, L.; Novo, M.; Perez-Gonzalez, C.; Samuel, I. D. W.; Leake, M. C.; Johnson, S.; Al-Soufi, W.; et al. Unveiling the Multi-Step Solubilization Mechanism of Sub-Micron Size Vesicles by Detergents. *Scientific Reports* **2019**, *9*.

(2) Juan-Colas, J.; Dresser, L.; Morris, K.; Lagadou, H.; Ward, R. H.; Burns, A.; Tear, S.; Johnson, S.; Leake, M. C.; Quinn, S. D. The Mechanism of Vesicle Solubilization by the Detergent Sodium Dodecyl Sulfate. *Langmuir* **2020**, *36* (39), 11499-11507.

(3) Armstrong, J. K.; Wenby, R. B.; Meiselman, H. J.; Fisher, T. C. Vybrant (Tm) Dio, Dii and Did Dyes for Multiple Labeling of Red Blood Cell Populations for in Vivo Survival Studies. *Blood* **2004**, *104* (11), 442a-442a.

(4) Zhang, Z.; Yomo, D.; Gradinaru, C. Choosing the Right Fluorophore for Single-Molecule Fluorescence Studies in a Lipid Environment. *Biochim Biophys Acta Biomembr* **2017**, *1859* (7), 1242-1253.

(5) Flagmeier, P.; De, S. M.; Michaels, T. C. T.; Yang, X. T.; Dear, A. J.; Emanuelsson, C.; Vendruscolo, M.; Linse, S.; Klenerman, D.; Knowles, T. P. J.; et al. Direct Measurement of Lipid Membrane Disruption Connects Kinetics and Toxicity of a Beta 42 Aggregation. *Nature Structural & Molecular Biology* **2020**, *27* (10), 886.

(6) De, S.; Wirthensohn, D. C.; Flagmeier, P.; Hughes, C.; Aprile, F. A.; Ruggeri, F. S.; Whiten, D. R.; Emin, D.; Xia, Z.; Varela, J. A.; et al. Different Soluble Aggregates of Abeta42 Can Give Rise to Cellular Toxicity through Different Mechanisms. *Nat Commun* **2019**, *10* (1), 1541.

(7) Miller, H.; Cosgrove, J.; Wollman, A. J. M.; Taylor, E.; Zhou, Z.; O'Toole, P. J.; Coles, M. C.; Leake, M. C. High-Speed Single-Molecule Tracking of Cxcl13 in the B-Follicle. *Front Immunol* **2018**, *9*, 1073.

(8) Preus, S.; Noer, S. L.; Hildebrandt, L. L.; Gudnason, D.; Birkedal, V. Isms: Single-Molecule Fret Microscopy Software. *Nat Methods* **2015**, *12* (7), 593-594.

(9) Kamrath, R. F.; Frances, E. I. Mass-Action Model of Mixed Micellization. *J. Phys. Chem.* **1984**, *88* (8), 1642-1648.
