# Supplementary material for: Tween-20 Induces the Structural Remodeling of Single Lipid Vesicles
Source: J Phys Chem Lett. 2022 Jun 9;13(23):5341–50. doi: 10.1021/acs.jpclett.2c00704 (PMC9208007; doi:10.1021/acs.jpclett.2c00704)
Supplement: Supplementary file 2 — jz2c00704_si_002.pdf [file jz2c00704_si_002.pdf]

jz-2022-00704v.R1

Name: Peer Review Information for "Tween-20 Induces the Structural Remodeling of Single Lipid Vesicles"

First Round of Reviewer Comments

Reviewer: 1

Comments to the Author

The manuscript “Tween-20 induces the structural remodeling of single lipid vesicles” by Lara Dresser and colleagues describes important physical-chemical behavior of model lipid vesicles upon interaction with detergent Tween-20. The study is important and contributes novel knowledge about how lipid membranes (possibly biological membranes) respond to the addition of surfactants and (possibly) other lipid bilayer incorporating molecules, including those of natural origin.

The study was conducted by a team of scientist with lasting interest and extensive experience in understanding the physical-chemical effects underlying the behavior of model lipid membranes.

I find the study very detailed and informative; results are well presented and described; and the conclusions made are adequate. Multiple advantageous techniques and approaches were used and the results of all of them compared making the study methodologically very solid.

Based on all the above, I would recommend the manuscript to be published in The Journal of Physical Chemistry Letters after addressing the following points:

1. The Authors assume that initial interaction between lipid membrane and Tween-20 involves deposition of detergent molecules on the surface of the membrane. Is this the reason for vehicle size enlargement (could this lead to 75% vesicle size enlargement) or the enlargement is also due to penetration of detergent into the bilayer hydrophobic core?

2. Do Authors assume any cooperativity effect leading to pore formation in the bilayer due to detergent membrane remodeling – is there critical detergent-to-lipid ratio or detergent concentration above which the pores are formed or it is time-defined process?

3. The Authors bring the critical micelles concertation (CMC) of Tween-20 in several places in the manuscript. However, they observed Tween-20-induced changes in lipid vesicles morphology even below the CMC. Then, how CMC could be important for the processes described in the manuscript?

Reviewer: 2

Comments to the Author

Dresser et al. sought to understand the underlying mechanisms of Tween-20 mediated structural remodelling of ~200 nm LUVs in both freely diffusing and surface-immobilized states. The authors used a combination of biophysical methods - QCM-D, DLS, FRET, Single vesicle imaging - to elucidate the stepwise solubilisation mechanism of individual lipid vesicles which is crucial to understanding membrane damage and permeabilisation. This is an important area of biophysical research, and the findings are of high impact. The delineation and impact of membrane restructuring and consequent disruption by surfactants like Tween-20 is a critical question in multiple biological processes. This foundational knowledge has implications for membrane trafficking, surfactant induced cytotoxicity, and drug delivery.

The data is convincing, and the authors' interpretation of the results matches the data presented. The authors can successfully determine stepwise changes in membrane upon addition of Tween-20 and uncover the underlying solubilisation mechanism. They demonstrate how Tween-20 modifies the structure and integrity of individual vesicles via multistep pathways involving mass gain, followed by membrane swelling and destabilisation and finally, content exchange before lysis. Statistical analysis and critical control experiments were provided. The use of low concentrations of Tween-20 below the CMC is innovative and determines the role of individual Tween-20 monomers in POPC membrane swelling and permeabilisation. This is striking because nonionic surfactants generally achieve membrane lysis once above their CMCs.

1. My biggest concern in this manuscript is Calcium ion mediated vesicle fusion for freely diffusing vesicles. It has been shown that vesicles can undergo fusion in the presence of calcium ions with or without the presence of protein assemblies (ACS Nano 2021, 15, 8, 12880–12887; PNAS 2020 117 (31) 18470-18476; Advanced Biosystem 2020, PMID: 33084207; BBA Biomembrane 2012, 1818, 695). I understand that the surface-tethered vesicles are spatially apart and could not fuse with each other. However, the freely diffusing vesicles may undergo fusion in the presence of calcium ions, which could appear as swelling and all the related changes. I wonder if it is possible to calcium-mediated vesicle fusion with Tween-20 induced membrane swelling. Another possibility is that authors can perform the same set of experiments with a different combination of reporter and ions e.g. Fluorescein and H+, and decouple calcium-mediated vesicle fusion with Tween-20 induced membrane swelling.

2. Based on these and previous results, it seems like the effect of Tween-20 is highly curvature dependent. Understanding the surfactant mediated membrane disruption for both LUVs (such synaptic/extracellular vesicles which carry cargos) and GUVs (similar or close to the size of the cells) is highly important. The interactions of surfactants with GUVs/living cells are of considerable interest, especially regarding cellular toxicity. However, the authors mention that 'A direct comparison between our findings on LUVs and the GUVs used previously is not straightforward due to variations in composition'. I wonder if it is possible to make large and small vesicles with the same composition and direct comparison to show how the curvature affects the Tween-20 mediated lipid membrane destabilisation.

3. The dynamical process of surfactant action in single cells and single LUVs and GUVs are still unexplored mainly because we cannot track single surfactant molecules in situ. Would it be possible to label Tween-20 with a dye and visualize the dynamics and how it affects the membrane insertion, mass gain, swelling and ultimately permeabilisation below and above CMC. This might be out of scope for this study, but in my opinion, it would be very interesting.

4. ITC based experiments to study transfer enthalpies between detergent and lipid below and above CMC also could be helpful to determine surfactant-membrane partitioning equilibria and solubilisation curves.

Author's Response to Peer Review Comments:

Point-by-Point Response to Reviewer Comments

Author Response to Comments by Reviewer 1

General Comments: *"The manuscript "Tween-20 induces the structural remodeling of single lipid vesicles" by Lara Dresser and colleagues describes important physical-chemical behavior of model lipid vesicles upon interaction with detergent Tween-20. The study is important and contributes novel knowledge about how lipid membranes (possibly biological membranes) respond to the addition of surfactants and (possibly) other lipid bilayer incorporating molecules, including those of natural origin. The study was conducted by a team of scientist with lasting interest and extensive experience in understanding the physical-chemical effects underlying the behavior of model lipid membranes. I find the study very detailed and informative; results are well presented and described; and the conclusions made are adequate. Multiple advantageous techniques and approaches were used and the results of all of them compared making the study methodologically very solid. Based on all the above, I would recommend the manuscript to be published in the Journal of Physical Chemistry Letters after addressing the following points."*

We thank Reviewer 1 for these positive and encouraging comments and for taking the time to carefully and critically review our manuscript. By leveraging major advances in model membrane biochemistry with highly-interdisciplinary biophysical approaches, we have interrogated a critically important interaction, and revealed key mechanistic details that are otherwise obscured by the ensemble average. We envisage that the presented tools and techniques will be widely applicable beyond the surfactant-vesicle interactions studied here and be of substantial interest to a broad readership of *J. Phys. Chem. Lett*. As we mentioned in the original version of the manuscript, and as Reviewer 2 also points out, the foundational knowledge presented in our study has major implications for understanding membrane trafficking, detergent-induced cytotoxicity, drug delivery, and membrane damage events induced by disruptive agents with important biomedical significance.

Comment 1: *The Authors assume that initial interaction between lipid membrane and Tween-20 involves deposition of detergent molecules on the surface of the membrane. Is this the reason for vesicle size enlargement (could this lead to 75% vesicle size enlargement) or the enlargement is also due to penetration of detergent into the bilayer hydrophobic core?*

We thank Reviewer 1 for raising this point. As demonstrated previously (Lichtenberg et al, *Biophys. J.*, 105, 289-299, 2013; Mrówczyńska et al, *Cell. Biol.,* 35, 991-995, 2011), the binding of the surfactant to the outer monolayer likely results in vesicle instability because of (i) a mass imbalance between the outer and inner monolayer and (ii) penetration of the curvophilic surfactant into the bilayer. With respect to the latter, the highly positive spontaneous curvature of Tween-20 is thus likely to affect bilayer bending and lead to the observed swelling behaviour. To clarify, we have now added the following text to the discussion section of the manuscript as follows (changes are shown in red text):

"Owing to its bulky headgroup and pliable hydrocarbon chain, Tween-20 can be modelled as a cone with positive spontaneous curvature, and thus we attribute the observed structural changes to vesicle instability induced by a mass imbalance between the outer and inner monolayers upon surfactant binding and penetration of the surfactant into the bilayer, the latter of which may lead to bilayer bending and the observed swelling behaviour(20,57)."

To support our assertions, we have also included the following citation to the revised manuscript:

57. Mrowczynska L, Salzer U, Iglic A, Hagerstrand H. Curvature factor and membrane solubilization, with particular reference to membrane rafts. Cell Biol Int. 2011;35(10):991-5.

Comment 2: *Do Authors assume any cooperativity effect leading to pore formation in the bilayer due to detergent membrane remodeling – is there critical detergent-to-lipid ratio or detergent concentration above which the pores are formed or it is time-defined process?*

We thank Reviewer 1 for this query. We may to some degree speculate how the change in detergent-to-lipid ratio affects pore formation. We found that Tween-20 and the LUV lipids do mix in the membrane, but with a limited compatibility: we found a Flory interaction parameter χ=1.2 (χ=0 indicates ideal miscibility, while χ =2 leads to phase separation). This limited miscibility suggests favourable interactions between Tween-20 monomers within the membrane. We speculate that the monomers may then dynamically cluster into structures that in turn locally disrupt the bilayer. However, as indicated by Reviewer 2, an alternative explanation for pore formation may be related to the change in membrane curvature as the vesicles swell. In our experiments, the composition and curvature change

simultaneously and decoupling these influences is an area of future work. In our revised manuscript we now include this discussion and recommendation for follow-up experimentation by inclusion of the following text:

"As χ=0 indicates ideal miscibility and χ>2 complete incompatibility, $\chi \approx$1.2 suggests favorable interactions between Tween-20 monomers within the membrane, and we speculate that the surfactant might dynamically cluster to locally disrupt the bilayer and lead to pore formation."

"While the dynamic clustering and insertion of Tween-20 into the bilayer may lead to local invaginations and permeabilization, an alternative explanation for the existence of pores may be related to bilayer bending as the vesicles swell. In all measurements discussed, the composition and curvature change simultaneously, and further work is required to decouple these influences."

Comment 3: *The Authors bring the critical micelles concertation (CMC) of Tween-20 in several places in the manuscript. How is the CMC important for the processes described in the manuscript?*

We thank Reviewer 1 for this comment. The CMC refers to the concentration at which the surfactant associates to form micelles with hydrophobic interiors and hydrophilic surfaces. Based on ensemble biophysical and thermodynamic approaches, many of which are not sensitive to short-lived and transient events, and as Reviewer 2 points out, non-ionic surfactants generally achieve membrane solubilization only once above their CMC. By contrast, our "single-vesicle" approaches reveal a striking result: substantial structural remodelling of intact POPC vesicles takes place at concentrations below the CMC, suggesting the role of Tween-20 monomers may be even more important for regulating membrane solubilization than first thought. To clarify this point, we have now included the following text into the revised version of the manuscript:

"Another aspect of our results that deserves attention is that low concentrations of Tween-20, below the CMC, produced substantial conformational changes. This not only suggests that individual Tween-20 monomers play a role in vesicle swelling and permeabilization, but is particularly striking because non-ionic detergents typically achieve membrane solubilization only once above their CMCs."

Author Response to Comments by Reviewer 2

General Comments: *"Dresser et al. sought to understand the underlying mechanisms of Tween-20 mediated structural remodelling of ~200 nm LUVs in both freely diffusing and surface-immobilized states. The authors used a combination of biophysical methods - QCM-D, DLS, FRET, Single vesicle imaging - to elucidate the stepwise solubilisation mechanism of individual lipid vesicles which is crucial to understanding membrane damage and permeabilisation. This is an important area of biophysical research, and the findings are of high impact. The delineation and impact of membrane restructuring and consequent disruption by surfactants like Tween-20 is a critical question in multiple biological processes. This foundational knowledge has implications for membrane trafficking, surfactant induced cytotoxicity, and drug delivery. The data is convincing, and the authors' interpretation of the results matches the data presented. The authors can successfully determine stepwise changes in membrane upon addition of Tween-20 and uncover the underlying solubilisation mechanism. They demonstrate how Tween-20 modifies the structure and integrity of individual vesicles via multistep pathways involving mass gain, followed by membrane swelling and destabilisation and finally, content exchange before lysis. Statistical analysis and critical control experiments were provided. The use of low concentrations of Tween-20 below the CMC is innovative and determines the role of individual Tween-20 monomers in POPC membrane swelling and permeabilisation. This is striking because nonionic surfactants generally achieve membrane lysis once above their CMCs."*

We also thank Reviewer 2 for taking the time to critically review our manuscript, and for providing encouraging and positive remarks. We agree that understanding the Tween-20 membrane interaction is a critical and timely problem, and as we mentioned in the original version of our manuscript, a major finding is that low concentrations of detergent, below the CMC, produce substantial and stepwise conformational changes. This finding was only achievable through the combination of highly interdisciplinary biophysical approaches, which we also agree and re-emphasize, are likely to have broad appeal and applications.

Comment 1: *My biggest concern in this manuscript is Calcium ion mediated vesicle fusion for freely diffusing vesicles. It has been shown that vesicles can undergo fusion in the presence of calcium ions with or without the presence of protein assemblies (ACS Nano 2021, 15, 8, 12880–12887; PNAS 2020 117 (31) 18470-18476; Advanced Biosystem 2020, PMID: 33084207; BBA Biomembrane 2012, 1818, 695). I understand that the surface-tethered vesicles are spatially apart and could not fuse with each other. However, the freely diffusing*

vesicles may undergo fusion in the presence of calcium ions, which could appear as swelling and all the related changes.

We thank Reviewer 2 for raising this important point, and we agree that under certain conditions, excess calcium can mediate fusion. Indeed, Allolio and Harries (ACS Nano, 2021, 15, 8, 12880-12887) and Schmid *et. al.* (Advanced Biosystems, 2020,4, 11, 2000153) report that negatively charged lipids, such as phosphatidylserine can undergo fusion in the presence of excess calcium without assistance from protein assemblies. In our case, and to avoid Ca^{2+} induced fusion, we worked with neutral lipids, which as Tahir et al (PNAS, 2020, 117, 31, 18470-18476) point out, were only observed to fuse when the vesicles encapsulatd nanoparticles and calcium was introduced.

To test explicitly whether our POPC vesicles fuse upon calcium addition, we performed a lipid mixing assay. Here, we mixed two populations of POPC LUVs in solution at a 1: 1 ratio. The first population contained only 0.1 % DiI (FRET donor), and the second 0.1 % DiD (FRET acceptor). As demonstrated previously (e.g. Diao et al, Nature Protocols, 2012, 7, 921-934), this system reports on vesicle fusion via an enhancement in FRET efficiency as lipids mix. Our results confirm that the addition of excess calcium to a 1: 1 mixture of POPC vesicles under the same buffer conditions and final lipid concentrations as those used in the Cal-520 ensemble experiments do not undergo fusion. This can be seen by the fact that there was no observable sensitized emission from the acceptor across the titration and that the apparent FRET efficiency remained invariant relative to its initial starting value in the absence of calcium.

It is also worth mentioning that the ensemble and single-vesicle FRET experiments, together with our EM, QCM-D, DLS and FCS analysis were performed without calcium present, and in all cases, we observed substantial levels of structural remodelling, all of which are supportive of Tween-20 induced swelling.

In response to this comment, we have now updated the manuscript to include: (i) additional text (shown in red below) describing the lipid mixing assay and results, (ii) a new Supplementary Figure to demonstrate that calcium does not induce vesicle fusion under the conditions tested and (iii) two additional citations.

Changes to the Text (additions shown in red):

"Control experiments indicated that (i) Ca^{2+} did not cross the membrane in the absence of Tween-20 (**Figure S7**) (ii) Ca^{2+} did not induce vesicle fusion, as has been observed previously for negatively charged vesicles (43, 44) (**Figure S8**) and (iii) the Cal-520 fluorescence is insensitive to Tween-20 (**Figure S9**)…"

New Supplementary Figure and Legend:

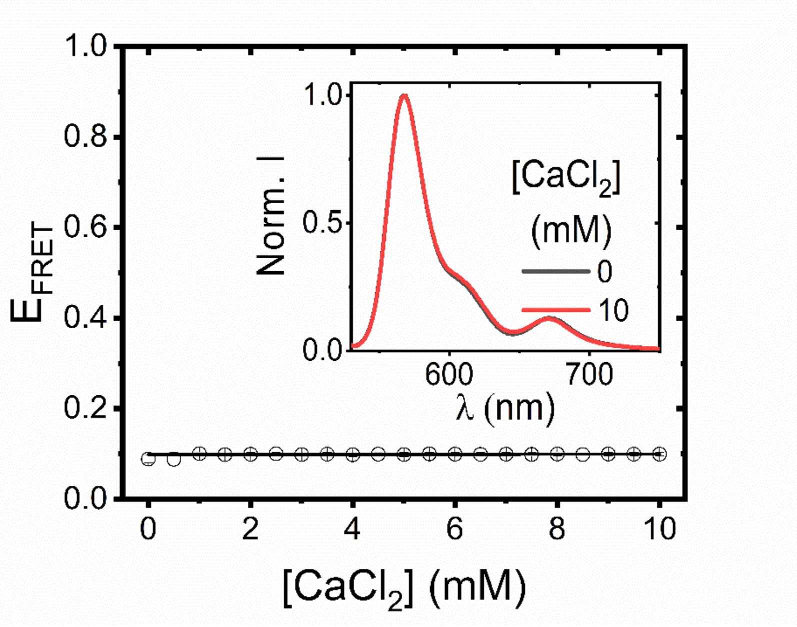

Figure S8. Ca^{2+} does not induce POPC vesicle fusion. Representative variation in E_{FRET} associated with two populations of POPC LUVs (one containing 0.1 % DiI (donor) and the other containing 0.1 % DiD (acceptor)) at a 1: 1 ratio in 50 mM Tris buffer (pH 8) in the absence and presence of $CaCl_2$. Inset: the corresponding normalized variation in fluorescence emission spectra (λ_{ex} = 520 nm) in the absence (black) and presence (red) of 10 mM $CaCl_2$.

New Citations:

43. Allolio C, Harries D. Calcium Ions Promote Membrane Fusion by Forming Negative-Curvature Inducing Clusters on Specific Anionic Lipids. ACS Nano. 2021.

44. Schmid YRF, Scheller L, Buchmann S, Dittrich PS. Calcium-Mediated Liposome Fusion to Engineer Giant Lipid Vesicles with Cytosolic Proteins and Reconstituted Mammalian Proteins. Adv Biosyst. 2020;4(11):e2000153.

Comment 2: *Based on these and previous results, it seems like the effect of Tween-20 is highly curvature dependent. Understanding the surfactant mediated membrane disruption for both LUVs (such synaptic/extracellular vesicles which carry cargos) and GUVs (similar or close to the size of the cells) is highly important. The interactions of surfactants with GUVs/living cells are of considerable interest, especially regarding cellular toxicity. However, the authors mention that 'A direct comparison between our findings on LUVs and the GUVs used previously is not straightforward due to variations in composition'. I wonder if it is possible to make large and small vesicles with the same composition and direct comparison to show how the curvature affects the Tween-20 mediated lipid membrane destabilisation.*

We thank Reviewer 2 for raising this point, and we agree that the interaction with Tween-20 and GUVs is also critically important. Indeed, our work was informed by previous literature in this area, and in the introduction of our manuscript we pointed towards several studies which have led to important structural insights during the interaction between Tween-20 and GUVs of varying size. A key advantage of the model membrane vesicles that we did not fully convey in the original version, is that their size, composition, content, radius of curvature and phase can be easily tuned and controlled, offering a platform from which to interrogate a huge matrix of vesicles against Tween-20 induced solubilization. To take this, and the reviewer's comment into account, we have now adapted our model-membrane preparation procedure and directly explored the interaction of 1 μm sized GUVs of identical composition to the LUVs against Tween-20. Specifically we prepared GUVs composed of 99.8 % POPC, 0.1 % DiI and 0.1 % DiD and explored their interaction with Tween-20 using (i) FRET spectroscopy and (ii) time-correlated single photon counting.

As the amount of Tween-20 was progressively increased into the GUV sample, similar trends to the LUVs were observed at 21°C. First, we observed a similar increase in DiI emission with a simultaneous decrease in DiD emission, which gave rise to an overall reduction in the apparent FRET efficiency across the titration. The general trend was similar to those reported in Figure 2B, however, while the Hill coefficient was similar to that obtained for the LUVs, the half-maximal concentration constant of the decay (0.13 ± 0.01 mM) was approximately double that observed for the LUVs (0.06 mM), indicating, as the reviewer suggests, membrane curvature may be a key regulator of the interaction. The interaction parameter also displayed a much smaller value (χ = 0.10 ± 0.02), also pointing towards a curvature dependence on miscibility. We also independently evaluated the fluorescence lifetime of DiI in the presence of DiD and here, the amplitude-weighted average lifetime progressively increased with Tween-20 concentration (an observation also observed from the LUVs) and again, the half-maximal concentration constant shifted towards higher values. Taken together, this new data allows us to directly compare LUVs and GUVs of identical composition against Tween-20, but more generally, opens up exciting opportunities from which to explore the effect of vesicle size, composition and phase on the Tween-20 induced membrane solubilization pathway, and this will be a major focus of future work.

In response to this comment, we have now updated the manuscript to include: (i) additional text (shown in red below) which describes this new data and quantitatively compares results obtained from LUVs with those returned from GUVs and (ii) a new Supplementary Figure showing representative changes to the apparent FRET efficiency and variations in DiI lifetime obtained from GUVs.

Changes to the Text (additions shown in red):

"Nevertheless, the vesicle size can, be easily tuned, and when 1 μm sized GUVs composed of 99.8 % POPC, 0.1 % DiI and 0.1 % DiD were studied in the presence of Tween-20, under identical conditions to those shown in **Figure 2B**, we observed common solubilization attributes: first, an overall reduction in FRET efficiency, and second, an enhancement of the amplitude-weighted average DiI lifetime as the concentration of Tween-20 was progressively increased (**Figure S14**). An important observation however is that the half-maximal concentration constant obtained for GUVs (~ 0.13 mM) was double that observed for the LUVs, and the interaction parameter was much smaller (χ ~ 0.1) indicating that membrane curvature may be a key regulator of the interaction. Overall, GUVs and sub-micron sized LUVs share similar solubilization attributes including nanoscale partitioning of lipids and the presence of a solution exchange step attributed to permeabilization."

New Supplementary Figure and Legend:

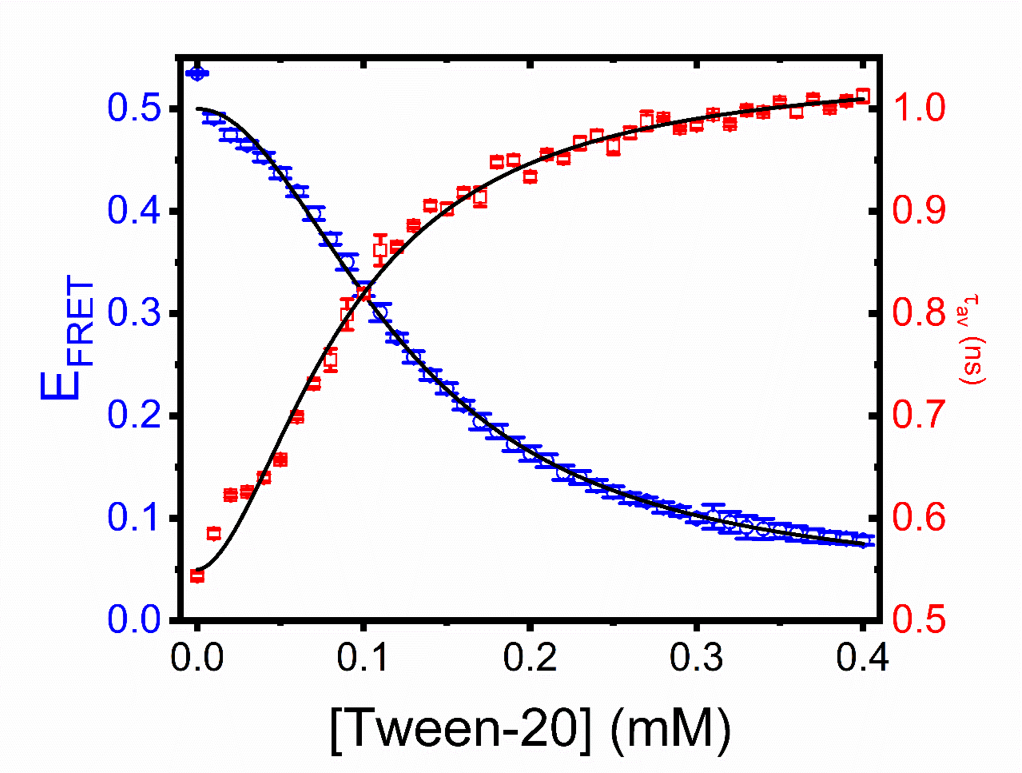

Figure S14. Interaction between Tween-20 and 1 μm sized GUVs monitored by ensemble FRET spectroscopy and time-correlated single photon counting. Variation in the ensemble FRET (blue) efficiency of 1 μm sized GUVs composed of 99.8 % POPC, 0.1 % DiI and 0.1 % DiD as a function of Tween-20 concentration. Also shown is the representative variation in DiI lifetime across the titration (red). Solid black lines correspond to Hill Model fits. Solution conditions: 25 μM POPC, 0.025 μM DiI, 0.025 μM DiD, 50 mM Tris, pH 8, 21°C.

Comment 3: ***The*** *dynamical process of surfactant action in single cells and single LUVs and GUVs are still unexplored mainly because we cannot track single surfactant molecules in situ. Would it be possible to label Tween-20 with a dye and visualize the dynamics and how it affects the membrane insertion, mass gain, swelling and ultimately permeabilisation below and above CMC? This might be out of scope for this study, but in my opinion, it would be very interesting.*

We thank Reviewer 2 for raising this point, and we agree that monitoring single detergent molecules interacting dynamically with the membrane surface at the earliest stages of solubilization is highly desirable. Indeed, our QCM-D analysis points towards an initial time-dependent mass gain and increase in vesicle viscoelasticity as detergent molecules are introduced. The initial deposition of Tween-20 onto the vesicle surface thus precedes major structural remodelling and lysis. However, to the best of our knowledge, there are currently no commercially available fluorescently-tagged Tween-20 derivatives on the market, nor has any detergent been successfully labelled and published to date. While some detergents have been reported to be intrinsically fluorescent, the labelling of Tween-20 is an open area of research, and as such, the proposed experiments would require overcoming this major synthesis hurdle. We speculate that it may be possible to introduce aldehydes and/or ketones into the Tween-20 structure which may then be modified with a probe, however, in the short-term, this is out of scope of the current study. Nevertheless, this is an important point, and certainly an area that should be investigated further. As such, we have added the following text to the manuscript to stimulate new research into this area:

Changes to the Text (additions shown in red):

"While the current data do not report on the initial dynamics of single surfactants directly interacting with the membrane, and further work in this area is highly desirable, it is notable that these events cannot be distinguished using a stand-alone technique, but rather they have emerged by utilizing a multidisciplinary toolkit."

Comment 4: *ITC based experiments to study transfer enthalpies between detergent and lipid below and above CMC also could be helpful to determine surfactant-membrane partitioning equilibria and solubilisation curves.*

We thank Reviewer 2 for this suggestion, and we agree that ITC, and other biophysical approaches, offer important insights into the Tween-20-membrane interaction. Indeed, such approaches have been performed extensively recently, for example to evaluate the CMC of Tween-20 (e.g. Smith et al, *Colloids and Surfaces B: Biointerfaces*, 211, 112320, 2022; Knoch et al, *Mol. Pharmaceutics*, 18, 3147-3157, 2021) and to characterize the initial interaction thermodynamics between surfactants, including Tween-20, and model membranes (e.g. Heerklotz & Seelig, *Biochm. Biophys. Acta.* 1508, 69,85, 2000; P. -H. Peuch et al. *Phys. Rev. Lett.* 90, 128304, 2003; Riske et al, *Biophys. Rev.* 9, 649-667, 2017; Chabanon & Rangamani, *Biochim. Biophys. Acta- Biomembranes.* 1860, 2032-2041, 2018). However, such approaches, rely on ensemble averaging across the entire system and unfortunately, ITC does not report on the structural remodelling of vesicles during the interaction, which makes decoupling vesicle remodelling from initial detergent-membrane thermodynamics a major barrier. By contrast, an advantage of our FRET-based approach is that thermodynamic parameters (including a surfactant-to-lipid binding energy and free energy of mixing) can now be assigned to the interaction via application of a mass-action model to the solubilization curves shown in Figure 2B. Furthermore single vesicles can now be interrogated on a vesicle-by-vesicle basis, which bypasses limitations associated with conventional ensemble averaging tools.

To clarify and highlight the advantages of the FRET approach relative to techniques such as ITC, we have now included the following additional text and citations in the revised manuscript:

"Thermodynamic approaches, including isothermal titration calorimetry, have also been employed extensively to evaluate the detergent CMC(18,19), and to characterize the thermodynamics of the surfactant-membrane interaction(11, 20-22). However, such approaches rely on ensemble averaging across the entire system and cannot report on individual vesicle conformation."

"From these features we extracted thermodynamic information about the surfactant-lipid interactions by adopting a mass-action model (**Supplementary Text 1, Figure S2**)."

"However, it is worth re-emphasizing that key advantages of the FRET-based approach are that thermodynamic parameters can be assigned to the surfactant-membrane interactions via application of a mass-action model and fluorescently-tagged vesicles can be interrogated on a vesicle-by-vesicle basis bypassing major limitations associated with ensemble averaging."

Additional citations:

18. Smith OEP, Waters LJ, Small W, Mellor S. CMC determination using isothermal titration calorimetry for five industrially significant non-ionic surfactants. Colloid Surface B. 2022;211.
19. Knoch H, Ulbrich MH, Mittag JJ, Buske J, Garidel P, Heerklotz H. Complex Micellization Behavior of the Polysorbates Tween 20 and Tween 80. Mol Pharm. 2021;18(8):3147-57.
20. Heerklotz H, Seelig J. Titration calorimetry of surfactant-membrane partitioning and membrane solubilization. Biochim Biophys Acta. 2000;1508(1-2):69-85.
21. Puech PH, Borghi N, Karatekin E, Brochard-Wyart F. Line thermodynamics: adsorption at a membrane edge. Phys Rev Lett. 2003;90(12):128304.
22. Riske KA, Domingues CC, Casadei BR, Mattei B, Carita AC, Lira RB, et al. Biophysical approaches in the study of biomembrane solubilization: quantitative assessment and the role of lateral inhomogeneity. Biophys Rev. 2017;9(5):649-67.

In addition to the above revisions, the following non-scientific changes were made to the revised manuscript:

- The title in the main manuscript file and Supporting Information is now set in case, with the first letter of each principal word capitalized.
- The abstract is now labelled in the main manuscript.
- The TOC graphic has been resized to 2 in x 2 in and placed on the same page as the abstract.
- References in both the main file and supporting information have been formatted for compatibility with *J. Phys. Chem. Lett.*
- The acknowledgements section has been updated to thank both reviewers for critically evaluating our manuscript and for providing positive and constructive comments.

jz-2022-00704v.R2

Name: Peer Review Information for "Tween-20 Induces the Structural Remodeling of Single Lipid Vesicles"

Second Round of Reviewer Comments

Reviewer: 2

Comments to the Author

The authors have addressed all the comments in the revised version and improved the quality of the manuscript. I found the revised manuscript suitable for publication in JPCL.

There are few typos in the manuscript. That should be checked once more carefully -

'.. when 0.04 mM and 0.06 mM Tween-20 was flushed...'

was should be were

'..followed by substantial mass loss to the solution…'

should be '…followed by a substantial mass loss to the solution'

'….remodelling event that precedes the loss of material to solution.'

Should be '….remodelling event that precedes the loss of material to the solution.'

'….changes in the fluorescence emission spectra after addition of…'

should be '…changes in the fluorescence emission spectra after the addition of..'

'…the acceptor emission and corresponding increase in the ..'

should be '…the acceptor emission and a corresponding increase in the ...'

'…PC vesicles composed of modest cholesterol content resists'

Should be '…PC vesicles composed of modest cholesterol content resist..'

Fig caption 'Histograms of the mean FRET efficiency obtained from ….'

Should be 'Histograms of the mean FRET efficiency were obtained from….

'DLS and FCS and further confirms Tween-20…'

Should be' DLS and FCS and further confirms that Tween-20…'

‘fluorescence and QCM-D data, the results are broadly supportive of Tween-20…’

should be ‘fluorescence and QCM-D data, and the results are broadly supportive of Tween-20…’

Reviewer: 1

Comments to the Author

The Authors addressed all my comments appropriately. Therefore, I recommend the manuscript for publication.

Author's Response to Peer Review Comments:

We have now updated the supporting information paragraph in the main file, numbered the supporting information pages appropriately, and corrected minor typos noted by Reviewer 2.
